# Arabidopsis WSS1A, a DNA‐protein crosslink repair protease, delays leaf senescence in cooperation with SUMO3

**DOI:** 10.1111/tpj.71094

**Published:** 2026-08-22

**Authors:** Sanghoon Park, Hyunwoo Oh, Ukcheol Jeong, Jae Ho Lee, Hyeyoung Choi, Hyunsu Park, Jinkwang Kim, Yongmin Kim, Junmin Kwak, Yeong Seon Yoon, Zhonghai Li, Jong‐Chan Lee, Hye Ryun Woo

**Affiliations:** ^1^ Department of New Biology Daegu Gyeongbuk Institute of Science and Technology (DGIST) Daegu 42988 Korea; ^2^ Korean Bioinformation Center (KOBIC) Korea Research Institute of Bioscience and Biotechnology (KRIBB) Daejeon 34141 Korea; ^3^ Baekdudaegan Global Seed Vault (BGSV) Korea Arboreta and Gardens Institute (KoAGI) Bonghwa‐gun 36209 Korea; ^4^ State Key Laboratory of Tree Genetics and Breeding College of Biological Sciences and Technology, Beijing Forestry University Beijing 100083 China; ^5^ New Biology Research Center Daegu Gyeongbuk Institute of Science and Technology (DGIST) Daegu 42988 Korea

**Keywords:** leaf senescence, DNA damage, DNA‐protein crosslink, WSS1A, LLPS, SUMO

## Abstract

Leaf senescence, the final stage of leaf development, is regulated by complex interplays of intrinsic genetic programs and environmental cues. Throughout their lifetimes, all living organisms encounter various endogenous and environmental challenges, many of which can cause potentially fatal DNA damage. Among these, DNA‐protein crosslinks (DPCs) are particularly deleterious, as they obstruct essential processes such as replication and transcription, thereby compromising genome integrity and ultimately leading to premature aging across species. However, the biological significance of DPCs and their repair mechanisms in leaf senescence remains unexplored. Here, we demonstrate that cis‐platin (cis‐Pt), a potent DPC inducer, accelerates leaf senescence in Arabidopsis. We reveal that Arabidopsis DPC repair factor WSS1A, a WLM/Spr‐T metalloprotease, plays a negative role in leaf senescence induced by cis‐Pt treatment, darkness, and leaf age. WSS1A forms nuclear condensates via liquid–liquid phase separation both *in vitro* and *in vivo*, which is cooperatively driven by its N‐terminal segment and intrinsically disordered region. Mechanistically, WSS1A non‐covalently interacts with SMALL UBIQUITIN MODIFIER 3 (SUMO3) through its SUMO‐interacting motif and is also covalently SUMOylated by SUMO3. Genetic analysis further reveals that WSS1A and SUMO3 act in the same pathway to control cis‐Pt‐induced leaf senescence. Together, this study establishes a conceptual framework connecting DPC repair and SUMO3‐dependent regulation of WSS1A in modulating leaf senescence in Arabidopsis.

## Significance Statement

DNA‐protein crosslinks (DPCs) are highly deleterious lesions that impede transcription and DNA replication, yet whether their repair affects leaf senescence has remained unexplored. Here, we reveal that the evolutionarily conserved DPC repair protease WSS1A, which forms nuclear condensates via liquid–liquid phase separation and cooperates with SUMO3, negatively regulates leaf senescence in Arabidopsis, establishing DPC repair as a safeguard against premature senescence.

## INTRODUCTION

Leaf senescence is an age‐dependent process that involves the degradation of macromolecules, the systemic dismantling of cellular structures, as well as extensive transcriptional and epigenetic reprogramming (Brusslan et al., 2015; Lim et al., 2007; Woo et al., 2019). The initiation and progression of leaf senescence are primarily controlled by developmental age but are also strongly influenced by various endogenous signals, such as hormonal changes, and exogenous factors, including environmental stresses like drought, extreme temperatures, shading, and pathogen infections (Guo et al., 2021; Guo & Gan, 2012). Thus, leaf senescence is a highly regulated developmental process that enables plants to recycle nutrients from aging leaves to younger tissues, ensuring reproductive success and environmental adaptation essential for plant fitness (Avila‐Ospina et al., 2014; Lim et al., 2007; Woo et al., 2013).

Throughout their lifespan, living organisms inevitably suffer from various sources of DNA damage, arising endogenously from replication errors or metabolic by‐products such as reactive oxygen species and exogenous genotoxic stresses (Kimura & Sakaguchi, 2006; Szurman‐Zubrzycka et al., 2023). If left unrepaired, these damages disrupt essential cellular processes, including DNA replication and transcription, and can lead to mutations, growth retardation, and, ultimately, premature aging (Britt, 1999; Chakarov et al., 2014). Extensive studies in animals and plants have demonstrated that defects in DNA repair pathways accelerate aging, supporting the DNA damage theory of aging (Maynard et al., 2015). For instance, deficiencies in key DNA repair proteins, such as BReast CAncer gene 1 (BRCA1) and Ku70/Ku80, increase susceptibility to genomic instability and age‐related disorders in animals (Cao et al., 2003; Li et al., 2007, 2016). Similarly, Ataxia‐Telangiectasia Mutated (ATM) kinase suppresses DNA double‐strand break (DSB)‐induced leaf senescence by epigenetically repressing the expression of senescence‐associated genes (SAGs) through histone modification in Arabidopsis (Li et al., 2020).

DNA‐protein crosslinks (DPCs)—a highly cytotoxic form of DNA damage—covalently link proteins to DNA, which physically blocks replication and transcription, thereby leading to genomic instability (Barker et al., 2005). They arise through both enzymatic and non‐enzymatic mechanisms. Enzymatic DPCs occur as transient intermediates in essential DNA transactions, such as topoisomerase cleavage and DNA methylation (Nitiss, 2009a, 2009b; Pommier et al., 2014). However, under replication stress or exposure to certain chemicals (e.g., camptothecin [CPT], etoposide, and zebularine), these intermediates become stabilized, forming toxic DPC lesions (Enderle et al., 2019; Hurd et al., 1999; Li & Liu, 2001; Nitiss, 2009a, 2009b; Prochazkova et al., 2022). Non‐enzymatic DPCs result from covalent protein‐DNA crosslinking induced by exogenous agents such as formaldehyde, ionizing radiation, and UV light (Barker et al., 2005; Stingele et al., 2015, 2017). Although DPC accumulation is known to compromise cellular function and is linked to premature aging and neurodegeneration in mammals (Huang et al., 2020; Maskey et al., 2014; Zahn et al., 1999), its potential impact on plant senescence remains largely unexplored.

DPC repair relies on specialized mechanisms that are evolutionarily conserved across species. Proteolysis‐dependent DPC repair is a key pathway mediated by WLM/Spr‐T metalloproteases such as Weak suppressor of smt3 (Wss1) in yeast and SPRTN (also known as DVC1) in mammals (Lopez‐Mosqueda et al., 2016; Stingele et al., 2014, 2016). Especially, defects in SPRTN are associated with Ruijs‐Aalfs syndrome in humans, characterized by premature aging, highlighting the importance of efficient DPC repair (Lessel et al., 2014). Wss1 alleviates replication stress, a driver of replicative senescence in budding yeast (Maddi et al., 2020). In Arabidopsis, WSS1A plays a role in maintaining genome stability under both enzymatic DPC and non‐enzymatic DPC‐inducing conditions (Enderle et al., 2019). Plants lacking WSS1A exhibit hypersensitivity to DPC‐inducing agents (e.g., cis‐Pt and CPT), along with developmental abnormalities such as reduced root growth and fertility. WSS1A is also known to cooperate with other DNA repair pathways, including hydrolysis mediated by TYROSYL‐DNA PHOSPHODIESTERASE 1 (TDP1) and TDP2, as well as nuclease repair mediated by METHYL METHANESULFONATE AND UV‐SENSITIVE PROTEIN 81 (MUS81), to efficiently resolve DPCs (Enderle et al., 2019; Hacker et al., 2022). In addition, WSS1A prevents persistent DNA DSBs under replication stress in association with the non‐homologous end joining machinery (Hacker et al., 2021). Although these findings highlight the role of WSS1A in protecting plant cells under genotoxic stress conditions, its specific function in leaf senescence and the underlying molecular mechanisms need to be determined.

Liquid–liquid phase separation (LLPS) has emerged as a key mechanism in the dynamic organization of cellular components into membrane‐less compartments, enabling cells to efficiently regulate biological processes such as stress response and transcriptional regulation (Feng et al., 2021; Park et al., 2020; Sabari et al., 2018; Wang et al., 2021). LLPS also contributes to the spatiotemporal organization of DNA repair factors at sites of DNA damage (Alberti et al., 2019; Levone et al., 2021; Wang et al., 2023). For example, p53‐BINDING PROTEIN 1 (53BP1) in HeLa cells and RADIATION SENSITIVE 52 (RAD52) in yeast form phase‐separated condensates at DSB sites, facilitating repair efficiency (Oshidari et al., 2020; Pessina et al., 2019; Zhang et al., 2022). It was also reported that HMTase SU(VAR)3‐9‐RELATED 2 (SUVR2) in *Medicago truncatula* promotes DSB repair by recruiting RAD51 into biomolecular condensates (Liu et al., 2022). However, the involvement of LLPS in DPC repair has yet to be thoroughly investigated.

SMALL UBIQUITIN‐LIKE MODIFIER (SUMO) is a well‐known post‐translational modifier, covalently attached to target proteins to regulate their stability, localization, and/or activity. SUMOylation plays critical roles in various cellular processes, including transcription, stress responses, and cell‐cycle progression (Enserink, 2015; Vertegaal, 2022). SUMOylation is also essential for maintaining genome stability, particularly in resolving DPCs. For instance, SUMO ligases, such as SAP AND MIZ1 DOMAIN‐CONTAINING LIGASE 1 (SIZ1) in yeast and PROTEIN INHIBITOR OF ACTIVATED STAT PROTEIN 4 (PIAS4) in humans, SUMOylate topoisomerase‐induced DPCs (TOP1ccs and TOP2ccs) to promote their subsequent degradation. SUMOylated DPCs are subsequently marked for proteasomal degradation by SUMO‐targeted ubiquitin ligases, including the SYNTHETIC LETHAL OF UNKNOWN FUNCTION 5/8 (SLX5/8) complex in yeast and RING FINGER PROTEIN 4 (RNF4) in humans (Borgermann et al., 2019; Liu et al., 2021; Serbyn et al., 2021). In Arabidopsis, the STRUCTURAL MAINTENANCE OF CHROMOSOMES 5/6 (SMC5/6) complex, via its NON‐SMC ELEMENT 2 (NSE2) subunit, functions as an E3 SUMO ligase that promotes DPC SUMOylation, thereby facilitating repair and removal (Dvořák Tomaštíková et al., 2023). It is also known that yeast Wss1 and mammalian SPRTN cooperate with SUMOylation pathways to enhance DPC resolution (Balakirev et al., 2015; Mullen et al., 2010; Ruggiano et al., 2021). Nevertheless, the functional relationship between SUMOylation and WSS1A in the regulation of leaf senescence remains to be elucidated.

While the biochemical role of WSS1A in DPC repair is well‐established, its physiological involvement in leaf senescence and its potential functional coordination with the SUMOylation machinery have remained unexplored. To elucidate these unresolved questions, we investigated how WSS1A regulates leaf senescence, demonstrating its crucial role as a negative regulator of this process. Mechanistically, we found that WSS1A undergoes LLPS via the cooperative action of its N‐terminal segment (NTS) and intrinsically disordered region (IDR). Notably, WSS1A interacts with SUMO3 through its SUMO‐interacting motif (SIM). These findings provide novel insights into the molecular mechanisms by which WSS1A regulates leaf senescence, involving SUMOylation and LLPS.

## RESULTS

### Structural and functional conservation of Arabidopsis WSS1A in DPC‐proteolytic repair

In order to investigate how molecular regulators of DPC repair are involved in leaf senescence, we analyzed dark‐induced senescence phenotypes in Arabidopsis loss‐of‐function mutants of putative DPC repair components: DNA DAMAGE‐INDUCIBLE 1 (DDI1), MUS81, TDP1, WSS1A, and WSS1B whose transcripts are upregulated during late stages of leaf senescence (Guo et al., 2025). Among the mutants examined, the *wss1A‐1* mutant exhibited the most pronounced premature senescence phenotype (Figure S1), prompting us to further investigate the potential roles of WSS1A and its paralog WSS1B in regulating leaf senescence via DPC‐proteolysis repair.

**Figure 1:**
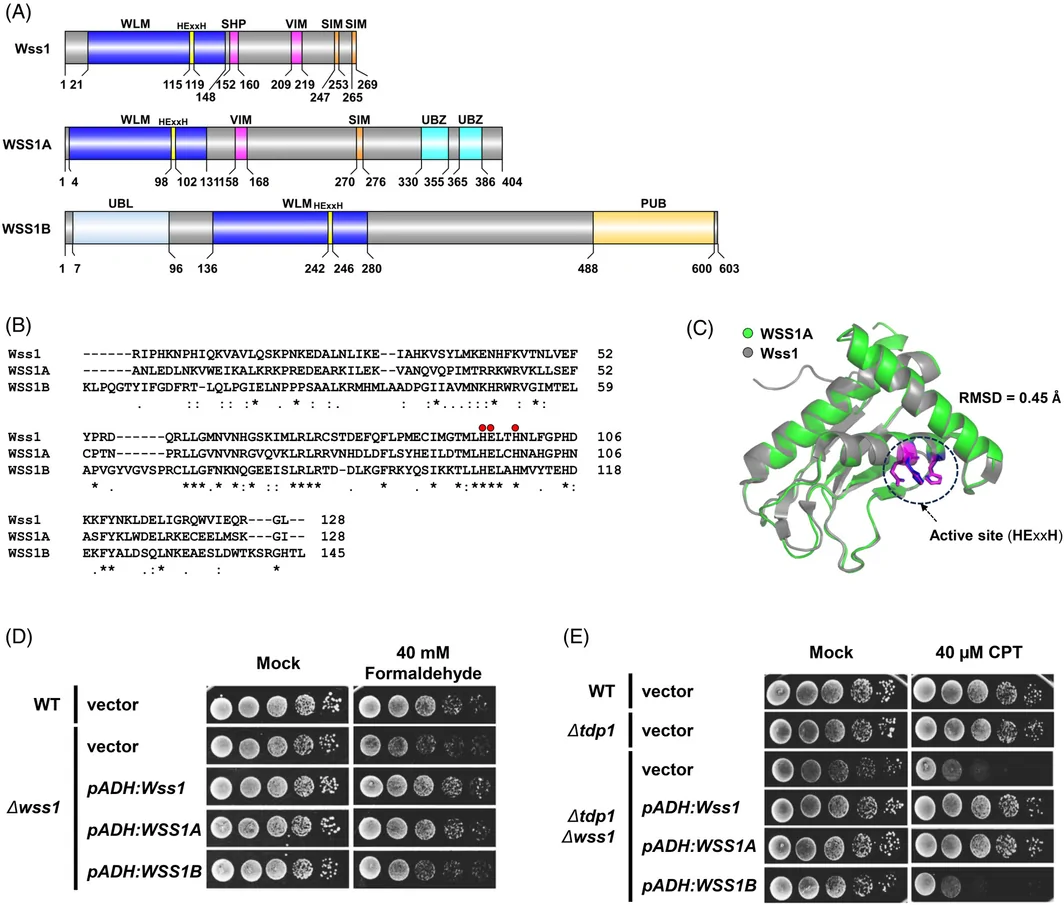
Functional conservation of Arabidopsis WSS1A and yeast Wss1 for DNA‐protein crosslink (DPC)‐proteolysis repair. (A) Domain architecture of the Arabidopsis WSS1A, WSS1B, and yeast Wss1 proteins. HExxH (H, histidine; E, glutamic acid; x, any amino acid), active site of WLM metalloprotease; PUB, associated with the ubiquitin‐proteasome system; SHP and VIM, CDC48‐binding motifs; SIM, SUMO‐interacting motif; UBL, ubiquitin‐like domain; UBZ, ubiquitin‐binding zinc‐finger domain; WLM, WLM protease domain. (B) Sequence alignment of the WLM domain of WSS1A, WSS1B, and Wss1 generated by Clustal Omega. The active site residues of WLM metalloprotease HExxH are marked by red circles. Asterisk (*), invariant residues; colon (:), highly similar residues; period (.), weak conservation between residues. (C) Structural comparison of the WLM domains of WSS1A and Wss1. Superposition of the predicted WLM structure of WSS1A (green; Alphafold3) and the crystal structure of Wss1 (gray; PDB:5XBN). Conserved catalytic residues (H–E–H) in the active sites of the two WLM domains are highlighted in blue (WSS1A) and magenta (Wss1). RMSD, root‐mean‐square deviation. (D, E) Yeast complementation assay. Fivefold serial dilutions of wild‐type (WT), *Δwss1*, *Δtdp1*, and *Δtdp1Δwss1* cells harboring an empty plasmid or plasmids containing *Wss1*, *WSS1A*, and *WSS1B* under the control of the *Alcohol Dehydrogenase* (*ADH*) promoter. Cells were treated with mock or 40 mM formaldehyde for 15 min, followed by two wash steps, and then spotted on SD/‐leucine (L) plates (D) or SD/‐L plates with or without 40 μM CPT (E). All plates were incubated for 2.5 days at 30°C.

To evaluate whether Arabidopsis WSS1A and WSS1B are functional orthologs to yeast Wss1, we first compared their domain architectures and conserved motifs. WSS1A and yeast Wss1 shared several key features, including the WLM zinc metalloprotease domain, the VIM (CELL DIVISION CYCLE 48 [CDC48]‐binding motif), and the SIM. Although WSS1A lacked the SHP motif, another CDC48‐binding motif present in Wss1, it instead contained two C‐terminal ubiquitin‐binding zinc‐finger (UBZ) domains (Figure 1A). By contrast, WSS1B retained the WLM domain but lacked both VIM and SIM, suggesting a potentially functional divergence.

Structure‐guided multiple sequence alignment mapped the WLM domain of WSS1A to residues 4–131 and that of WSS1B to residues 136–280 (Figure S2a,b), with strong conservation of the critical catalytic HExxH motif (where H is a histidine, E is a glutamic acid, and x is any amino acid) essential for DPC resolution (Figure 1B). Structural superimposition of AlphaFold3‐predicted models of WSS1A and WSS1B onto the yeast Wss1 crystal structure (PDB ID:5XBN, Reinking et al., 2020; Yang et al., 2017) revealed a high degree of topological similarity within the WLM domain, particularly at the proteolytic active site (Figure 1C; Figure S2c). This was further supported by low RMSD values (0.45 Å for WSS1A and 0.83 Å for WSS1B) (Figure S2c).

We next examined whether WSS1A and WSS1B could complement the function of yeast Wss1 in *Saccharomyces cerevisiae* under DPC‐inducing conditions. As reported (Stingele et al., 2014), the yeast strain lacking *Wss1* (*Δwss1*) exhibited defective growth following short exposure to formaldehyde, a non‐enzymatic DPC agent (Figure 1D). Constitutive expression of Arabidopsis *WSS1A* in the *Δwss1* background fully rescued the growth defect, whereas *WSS1B* overexpression failed to complement the formaldehyde hypersensitivity observed in *Δwss1* (Figure 1D). We further tested complementation under conditions generating Top1ccs using an enzymatic DPC‐inducing agent, CPT. Notably, overexpression of Arabidopsis WSS1A, but not WSS1B, restored CPT resistance in *Δtdp1 Δwss1* (Figure 1E). These findings demonstrate that WSS1A, not WSS1B, functions as a *bona fide* ortholog of yeast Wss1, capable of restoring both enzymatic and non‐enzymatic DPC repair defects.

### WSS1A negatively regulates dark‐induced and age‐dependent leaf senescence in Arabidopsis

**Figure 2:**
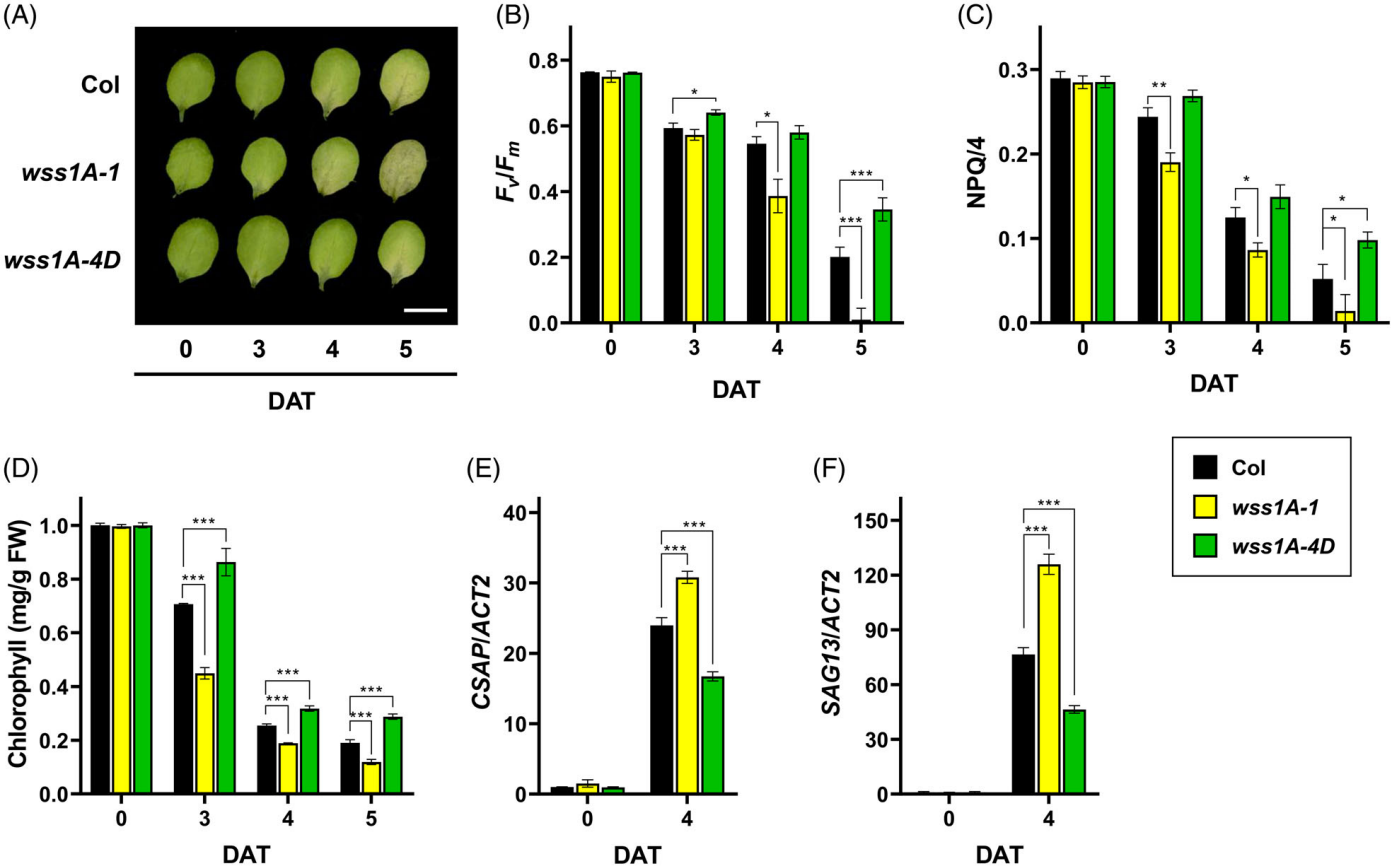
Dark‐induced leaf senescence phenotypes of *wss1A‐1* and *wss1A‐4D*. (A–D) Dark‐induced leaf senescence phenotypes of Col, *wss1A‐1*, and *wss1A‐4D* leaves at 0, 3, 4, and 5 DAT. Representative images (A) and changes in *F*_v_/*F*_m_ (B), non‐photochemical quenching divided by 4 (NPQ/4) (C), and chlorophyll content (mg g^−1^ fresh weight [FW]) (D) are shown. Scale bar, 1 cm. (E, F) Expression of *CSAP* (E) and *SAG13* (F) by RT‐qPCR. Data were normalized relative to *ACT2* and shown relative to the respective values in Col at 0 DAT. Data are presented as mean ± standard error (SEM) (*n* = 12 for B–D, *n* = 3 for E and F). *ACT2*, *ACTIN2*; *CSAP*, *CHLOROPLAST‐LOCALIZED SENESCENCE‐ASSOCIATED PROTEIN*; *SAG13*, *SENESCENCE‐ASSOCIATED GENE 13*. Asterisks indicate significant differences (*P < 0.05; **P < 0.01; ***P < 0.001, two‐way anova followed by Dunnett's multiple‐comparison test). All experiments were performed with at least three independent biological replicates, and representative results are shown.

To investigate the role of WSS1A in leaf senescence, we examined its involvement in dark‐induced and age‐dependent leaf senescence. We first assessed dark‐induced senescence by incubating detached third and fourth leaves of wild‐type Columbia (Col), *wss1A‐1* (loss‐of‐function allele; Enderle et al., 2019), and *wss1A‐4Dominant* (*wss1A‐4D*; gain‐of‐function allele; 14.6‐fold increase in *WSS1A* expression; Figure S3) plants under continuous darkness (Figure 2). At 5 days after dark treatment (DAT), *wss1A‐1* leaves exhibited pronounced yellowing compared to Col, whereas *wss1A‐4D* leaves were greener than Col (Figure 2A). These visual differences were supported by quantitative measurements of photosynthetic parameters. Col leaves at 5 DAT retained 26.3% of their initial photochemical efficiency (*F*_v_/*F*_m_) value, whereas *wss1A‐1* leaves showed a complete loss of *F*_v_/*F*_m_. By contrast, *wss1A‐4D* leaves maintained 45.3% of their initial *F*_v_/*F*_m_, indicating delayed senescence (Figure 2B). Consistently, non‐photochemical quenching (NPQ) and chlorophyll content were severely compromised in *wss1A‐1* leaves but significantly higher in *wss1A‐4D* leaves compared with Col (Figure 2C,D). The SAGs *CHLOROPLAST‐LOCALIZED SENESCENCE‐ASSOCIATED PROTEIN* (*CSAP*) and *SENESCENCE‐ASSOCIATED GENE 13* (*SAG13*) were strongly induced under continuous darkness at 4 DAT. Notably, both genes were induced to higher levels in *wss1A‐1* leaves, whereas *wss1A‐4D* leaves showed a markedly reduced induction compared with Col (Figure 2E,F).

We next evaluated the function of WSS1A in age‐dependent leaf senescence by monitoring the third and fourth leaves of Col, *wss1A‐1*, and *wss1A‐4D* plants from 12 to 28 days after emergence (DAE) at 8‐day intervals. Consistent with dark‐induced senescence results, the leaves of *wss1A‐1* and *wss1A‐4D* exhibited accelerated and delayed senescence phenotypes, respectively, relative to Col (Figure S4). At 28 DAE, *wss1A‐1* leaves showed pronounced yellowing, while *wss1A‐4D* leaves remained distinctively green (Figure S4a). Quantitatively, *F*_v_/*F*_m_ of *wss1A‐1* leaves dropped to 50.9% of the initial level, significantly lower than the 69.9% observed in Col, while *wss1A‐4D* leaves robustly maintained approximately 80% of the initial *F*_v_/*F*_m_ (Figure S4b). Similarly, NPQ and chlorophyll content declined more rapidly in *wss1A‐1* leaves but were better preserved in *wss1A‐4D* leaves throughout the examined leaf age (Figure S4c,d). Taken together, these findings demonstrate that WSS1A plays an important role in negatively regulating both dark‐induced and age‐dependent leaf senescence in Arabidopsis.

### Exogenous cis‐Pt treatment induces premature leaf senescence in Arabidopsis

DPC‐inducing agents are well‐established triggers of cellular senescence in mammalian systems (Chang et al., 1999; Han et al., 2002; Zhang et al., 2014). To investigate whether such agents promote leaf senescence in plants, we tested the effects of CPT, cis‐Pt, etoposide, and zebularine on senescence phenotypes in detached rosette leaves of Arabidopsis. Under our experimental conditions, only cis‐Pt reproducibly induced senescence phenotypes in detached leaves, such as decreased *F*_v_/*F*_m_ and NPQ (Figure S5), and was therefore selected for further analyses.

**Figure 3:**
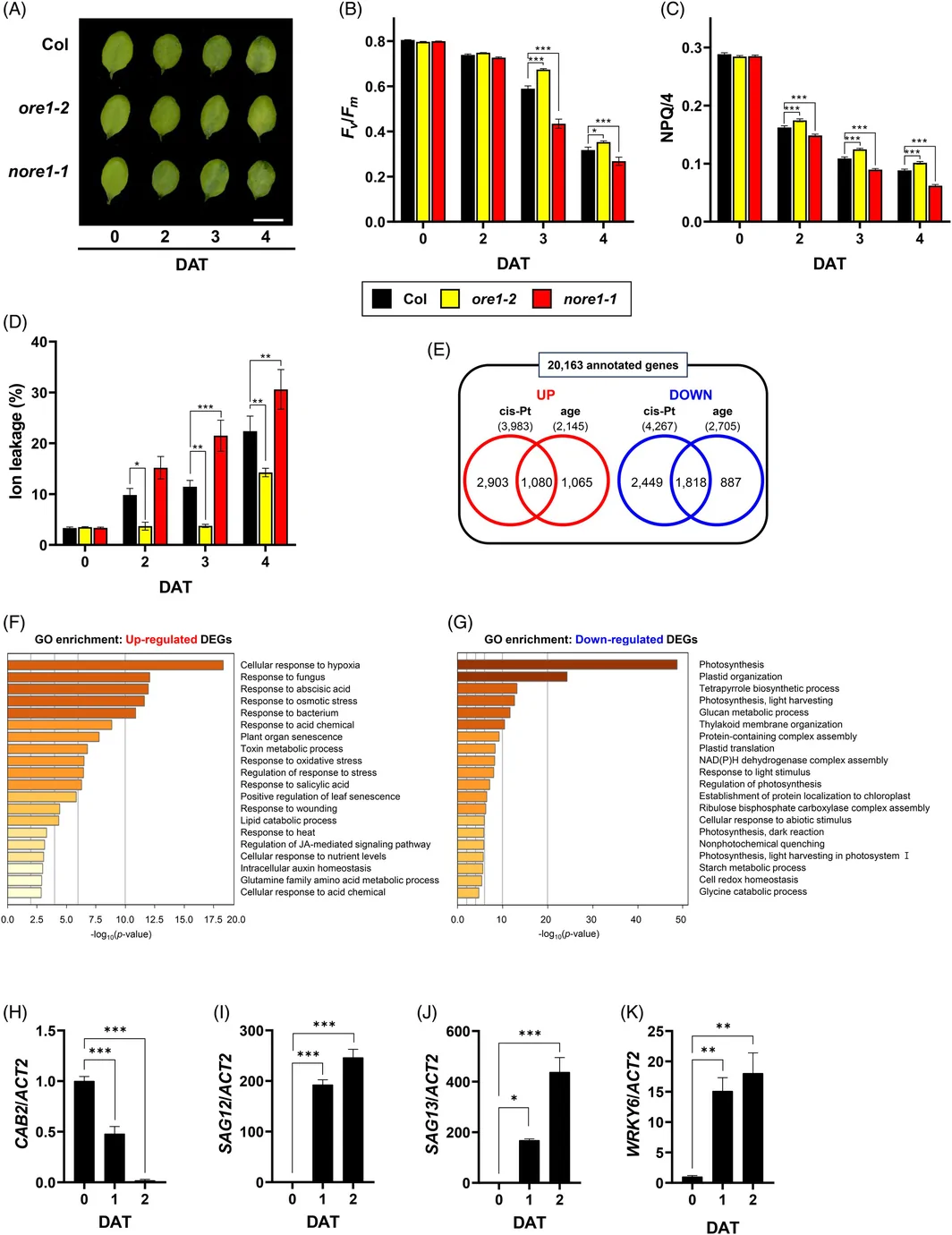
Premature leaf senescence induced by cis‐platin (cis‐Pt) in Arabidopsis. (A) Time‐course analysis of leaf senescence phenotypes induced by 15 μM cis‐Pt treatment in Col, *ore1‐2*, and *nore1‐1* leaves. Scale bar, 1 cm. (B–D) Changes in *F*_v_/*F*_m_ (B), NPQ/4 (C), and membrane ion leakage (D) in Col, *ore1‐2*, and *nore1‐1* leaves after 15 μM cis‐Pt treatment. Data are presented as mean ± SEM (*n* = 12). Asterisks indicate significant differences (*P < 0.05; **P < 0.01; ***P < 0.001, two‐way anova followed by Dunnett's multiple‐comparison test). All experiments were performed with at least three independent biological replicates, and representative results are shown. (E) Venn diagrams showing the overlap between cis‐Pt‐responsive differentially expressed genes (DEGs) (Data S1) and age‐dependent DEGs in Arabidopsis (Woo et al., 2016). The number of DEGs was determined using a threshold of |log_2_(FC)| >1.0 and *P* < 0.05 for both cis‐Pt treatment and for leaf age. (F, G) Gene ontology (GO) enrichment analysis of DEGs responsive to both cis‐Pt treatment and age in Arabidopsis leaves. The top 20 biological process clusters and their representative enriched terms are shown for upregulated (F) and downregulated (G) DEGs. (H–K) Transcript levels of *CAB2* (H), *SAG12* (I), *SAG13* (J), and *WRKY6* (K). 30 μM cis‐Pt was treated to Col leaves for the indicated days. Data were normalized relative to *ACT2* and shown relative to the respective values at 0 DAT. Data are presented as mean ± SEM (*n* = 3). *CAB2*, *CHLOROPHYLL A/B BINDING PROTEIN 2*. Asterisks indicate significant differences (*P < 0.05; **P < 0.01; ***P < 0.001, Student's *t*‐test). All experiments were performed with at least three independent biological replicates.

To determine the suitable concentration for assessing leaf senescence, we examined the dose‐dependent effects of cis‐Pt (7.5–60 μM) on detached leaves. Among the doses tested, 15 and 30 μM of cis‐Pt were selected for subsequent analyses as they provided optimal sensitivity for monitoring the progressive decline in physiological parameters, such as *F*_v_/*F*_m_ and NPQ (Figure S6). Application of 15 μM cis‐Pt to leaves of Col, a delayed senescence mutant *oresara1‐2* (*ore1‐2*; Kim et al., 2009), and an early senescence mutant *not oresara1‐1* (*nore1‐1*; Lee et al., 2016) resulted in distinct senescence responses. In Col leaves, cis‐Pt treatment induced characteristic senescence phenotypes such as a decrease in *F*_v_/*F*_m_ ratio, and NPQ, accompanied by increased membrane ion leakage (Figure 3A–D). Compared to Col, *ore1‐2* exhibited attenuated senescence responses, while *nore1‐1* showed more pronounced changes, indicating that cis‐Pt effectively causes premature leaf senescence in Arabidopsis.

To assess the similarities in the molecular programs between cis‐Pt‐induced and age‐dependent leaf senescence, we performed mRNA‐sequencing (mRNA‐seq) analysis of Col leaves treated with or without 30 μM cis‐Pt for 1 day and identified 8251 differentially expressed genes (DEGs) (Data S1). Comparison with a published age‐dependent transcriptome (Woo et al., 2016) revealed substantial overlap, with 27.1% of upregulated and 42.6% downregulated genes showing concordant expression changes (Figure 3E). Gene ontology (GO) enrichment analysis of overlapping DEGs showed significant enrichment of senescence‐ and stress‐associated pathways, including response to abscisic acid and salicylic acid and positive regulation of leaf senescence, among upregulated genes, whereas downregulated genes were enriched for photosynthesis‐related processes (Figure 3F,G). Consistently, senescence marker genes (*SAG12*, *SAG13*, and *WRKY6*) were strongly induced by cis‐Pt, while the photosynthetic gene *CHLOROPHYLL A/B BINDING PROTEIN 2* [*CAB2*] expression gradually declined (Figure 3H–K). Collectively, these observations demonstrate that cis‐Pt elicits physiological and transcriptional changes characteristic of leaf senescence, substantially overlapping with natural age‐dependent senescence programs in Arabidopsis.

### WSS1A is a key regulator of cis‐Pt‐induced leaf senescence in Arabidopsis

**Figure 4:**
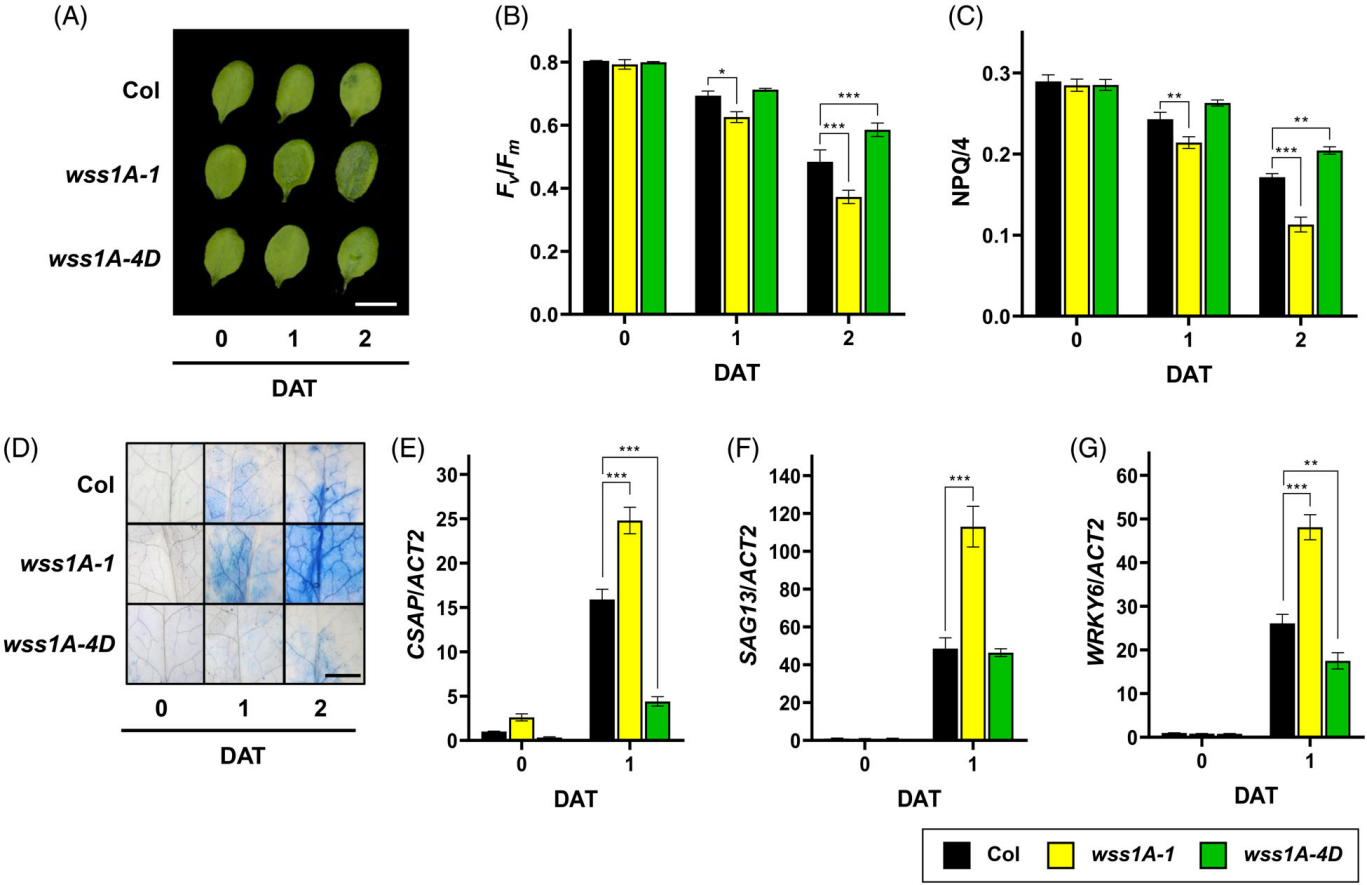
cis‐Platin (cis‐Pt)‐induced leaf senescence phenotypes of *wss1A‐1* and *wss1A‐4D*. (A–D) Time‐course analysis of leaf senescence phenotypes induced by 30 μM cis‐Pt treatment in Col, *wss1A‐1*, and *wss1A‐4D* plants at 0, 1, and 2 DAT. Representative images (A), *F*_v_/*F*_m_ (B), NPQ/4 (C), and trypan blue (TB) dye staining (D) are shown. Scale bar, 1 cm in (A) and 0.2 cm in (D). All experiments were performed with at least three independent biological replicates, and representative results are shown. (E–G) Expression of *CSAP* (E), *SAG13* (F), and *WRKY6* (G) by RT‐qPCR. Data were normalized relative to *ACT2* and shown relative to the respective values in Col at 0 DAT. Data are presented as mean ± SEM (*n* = 12 for B and C, *n* = 3 for E–G). Asterisks indicate significant differences (*P < 0.05; **P < 0.01; ***P < 0.001, two‐way anova followed by Dunnett's multiple‐comparison test).

Based on the findings above, we hypothesized that WSS1A functions as a negative regulator of cis‐Pt‐induced leaf senescence in Arabidopsis. To test this hypothesis, we treated the third and fourth leaves of Col, *wss1A‐1*, and *wss1A‐4D* with 30 μM cis‐Pt and monitored their senescence phenotypes. Following cis‐Pt treatment, *wss1A‐1* leaves exhibited pronounced premature senescence compared with Col leaves, whereas *wss1A‐4D* leaves remained green and intact at 2 DAT (Figure 4A). *wss1A‐1* and *wss1A‐4D* leaves showed significantly greater and lesser reductions, respectively, in physiological markers of leaf senescence, including *F*_v_/*F*_m_ ratio and NPQ compared with Col (Figure 4B,C). Additionally, *wss1A‐1* leaves displayed increased cell death, as evidenced by trypan blue (TB) staining, while *wss1A‐4D* leaves showed reduced cell death (Figure 4D). Consistent with these physiological observations, the expression levels of *SAGs* such as *CSAP*, *SAG13*, and *WRKY6* were significantly upregulated in *wss1A‐1* but attenuated in *wss1A‐4D* (Figure 4E–G). Thus, these results suggest that WSS1A plays a protective role in delaying cis‐Pt‐induced leaf senescence by participating in DPC repair.

### WSS1A undergoes LLPS both *in vitro* and *in vivo*

**Figure 5:**
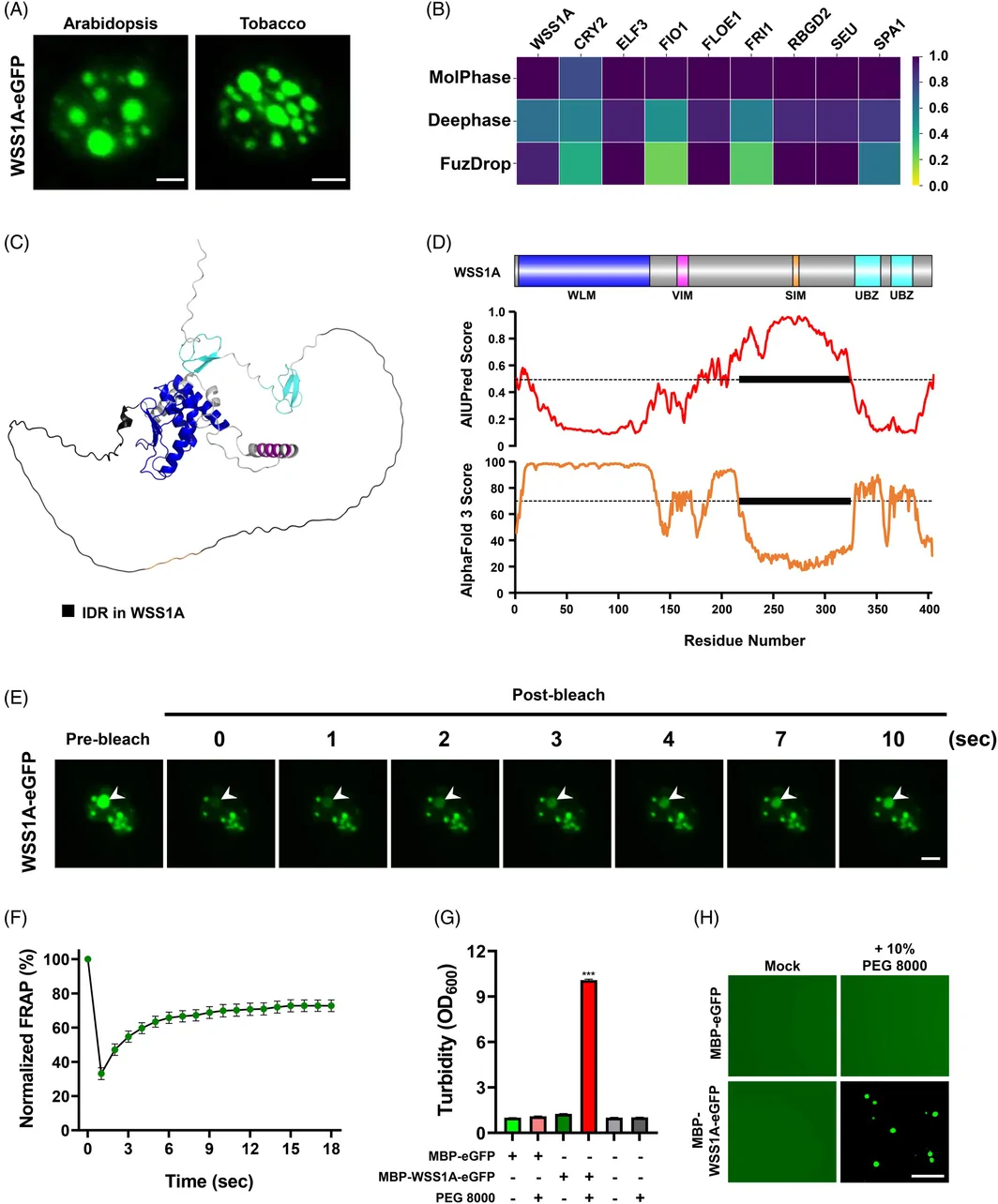
Liquid–liquid phase separation (LLPS) of WSS1A *in vivo* and *in vitro*. (A) Subcellular localization of transiently overexpressed WSS1A‐eGFP proteins in mesophyll protoplasts of Arabidopsis and leaves of *Nicotiana benthamiana*. Scale bar, 3 μm. (B) Phase separation propensity of WSS1A and known Arabidopsis LLPS proteins predicted using MolPhase, Deephase, and FuzDrop. CRY2, CRYPTOCHROME 2; ELF3, EARLY FLOWERING 3; FIO1, FIONA 1; FRI1, FRIGIDA 1; RBGD2, RNA‐BINDING GLYCINE‐RICH GROUP D 2; SEU, SEUSS; SPA1, SUPPRESSOR OF PHYA‐105 1. (C) Predicted 3D structure of WSS1A generated with AlphaFold3 and visualized using PyMOL. Black indicates the predicted IDR of WSS1A. IDR, intrinsically disordered region. (D) The disorder tendency of Arabidopsis WSS1A as predicted by AIUPred (upper) and AlphaFold 3 (lower). Black bar, predicted IDR. (E, F) Assessment of *in vivo* LLPS with transiently overexpressing WSS1A‐eGFP in Arabidopsis mesophyll protoplasts: representative images of WSS1A‐eGFP nuclear condensates in a mesophyll protoplast analyzed by fluorescence recovery after photobleaching (FRAP) experiments (E) and FRAP recovery plot of WSS1A‐eGFP nuclear condensates (F). The bleaching laser intensity was 100%, and the excitation wavelength was 488 nm. White arrowheads in (E) indicate photo‐bleached WSS1A‐eGFP condensates. The graph in (F) represents mean ± SEM (*n* = 10). FRAP experiments were performed with at least three independent biological replicates, and representative results are shown. Scale bar, 10 μm. (G, H) Assessment of *in vitro* LLPS with recombinant WSS1A‐eGFP proteins purified from *Escherichia coli*. Turbidity (G) and representative fluorescent images (H) of 10 μM MBP‐eGFP and MBP‐WSS1A‐eGFP proteins purified from *E*. *coli* in the presence and absence of 10% (w/v) PEG 8000 are shown. Data are presented as mean ± SEM (*n* = 3). Asterisks indicate significant differences (***P < 0.001, Student's *t*‐test). Scale bar, 5 μm.

To gain mechanistic insights into how WSS1A regulates leaf senescence and DPC repair, we first examined its subcellular localization. Transient overexpression of WSS1A‐eGFP (enhanced GREEN FLUORESCENCE PROTEIN) fusion protein in Arabidopsis mesophyll protoplasts and tobacco leaves revealed predominant localization to distinct nuclear puncta (Figure 5A). This distinct spatial organization was consistently observed in transgenic Arabidopsis plants overexpressing WSS1A‐eGFP. Notably, these puncta were preferentially enriched in lightly DAPI‐stained euchromatin regions, whereas they were excluded from DAPI‐dense chromocenters (Figure S7). These observations suggest that WSS1A puncta represent functional subnuclear compartments rather than non‐specific aggregates, potentially formed via LLPS. Consistently, *in silico* LLPS propensity predictions using MolPhase, DeePhase, and FuzDrop yielded high LLPS scores for WSS1A (0.99, 0.64, and 0.90, respectively), comparable with canonical Arabidopsis LLPS proteins (Figure 5B). Disorder prediction algorithms (AIUPred and AlphaFold 3) further identified an IDR (residues 216–323) (Figure 5C,D), indicating a structural predisposition for phase separation.

To verify whether WSS1A puncta correspond to liquid‐like condensates, we evaluated phase separation *in vivo*, fluorescence recovery after photobleaching (FRAP) experiments were performed in Arabidopsis mesophyll protoplasts transiently expressing WSS1A‐eGFP driven by the *CsVMV* promoter. When the puncta were bleached with a 488 nm laser with 100% intensity, the fluorescence intensity of WSS1A‐eGFP was quickly recovered within a time scale of seconds (Figure 5E,F). We then performed *in vitro* LLPS analysis with purified maltose‐binding protein (MBP)‐tagged WSS1A‐eGFP protein. Addition of 10% (w/v) polyethylene glycol 8000 (PEG 8000)—a molecular crowding agent known to induce phase separation (Andre & Spruijt, 2020)—induced an immediate increase in turbidity, as indicated by a rise in absorbance at 600 nm and formation of micron‐sized spherical droplets. By contrast, no droplets were observed in the absence of PEG (Figure 5G,H). Collectively, the results of both *in vivo* and *in vitro* experiments support the dynamic and liquid‐like nature of WSS1A condensates.

**Figure 6:**
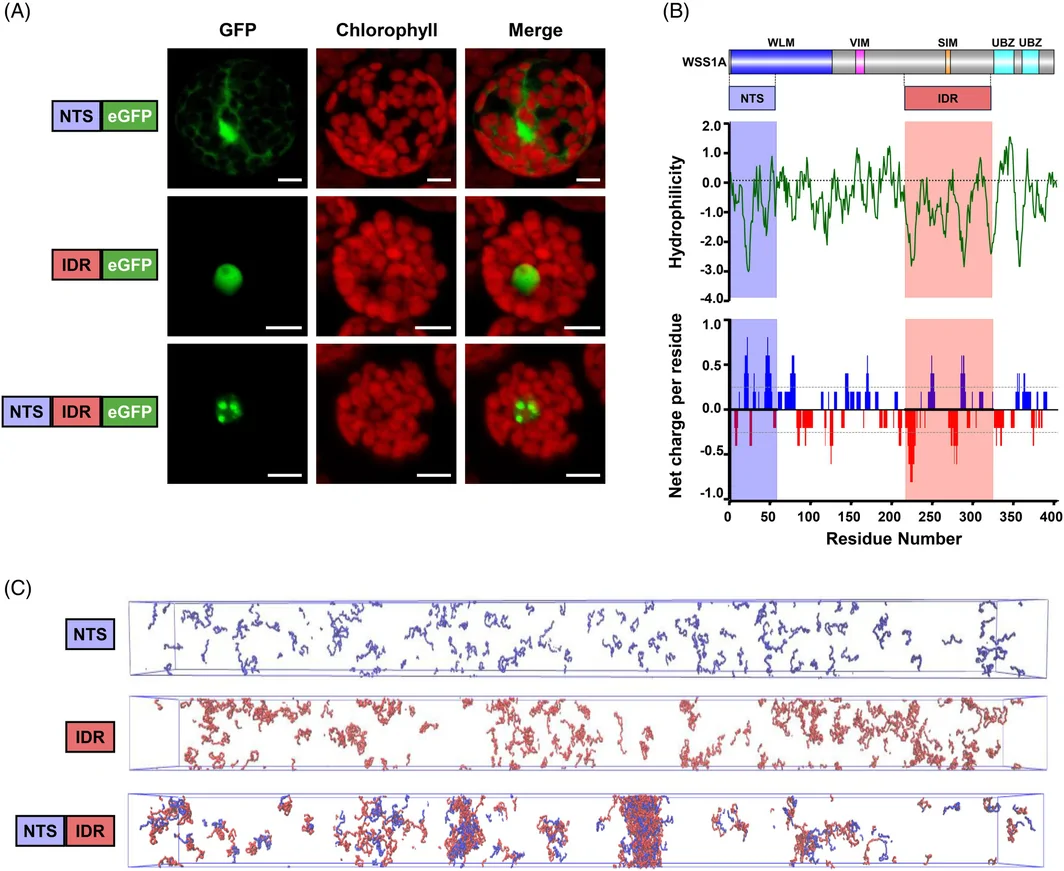
Contribution of the N‐terminal segment (NTS) and intrinsically disordered region (IDR) of WSS1A to nuclear condensate formation. (A) Subcellular localization and nuclear condensate formation of NTS, IDR, and NTS fused with IDR (NTS‐IDR) of WSS1A in the nucleus of Arabidopsis mesophyll protoplasts. Green, GFP signal; Red, autofluorescence for chlorophyll. Scale bar, 10 μm. NTS, N‐terminal segment. (B) Plots of the hydrophilicity and net charge per residue of WSS1A. Hydrophilicity and net charge per residue were calculated by ProtScale (Wilkins et al., 1999) and CIDER (Holehouse et al., 2017), respectively. (C) Slab simulations of 120‐molecule systems containing NTS, IDR, or NTS‐IDR of WSS1A proteins after 100 nanoseconds of coarse‐grained molecular dynamic simulation using GENESIS. Blue, NTS; Red, IDR.

Given the presence of an IDR (Figure 5C,D), we examined its contribution to LLPS. Contrary to our expectation, transient overexpression of IDR fused to eGFP (IDR‐eGFP) in mesophyll protoplasts resulted in diffuse nuclear localization without distinct puncta (Figure 6A, middle panel), indicating that the IDR alone was not sufficient for phase separation. Since LLPS often arises from multivalent interactions between proteins, including electrostatic and/or hydrophobic interactions (Lin et al., 2016; Nott et al., 2015; Yang et al., 2020), we analyzed charge distribution, hydrophilicity, and foldability using Classification of Intrinsically Disordered Ensemble Regions (CIDER), ProtScale, and FoldIndex (Holehouse et al., 2017; Prilusky et al., 2005; Wilkins et al., 1999). These analyses identified a distinct NTS (amino acids 1–57) enriched in positively charged and hydrophilic residues with a predominantly unfolded conformation (Figure 6B; Figure S8). Moreover, molecular dynamics (MD) simulations using the generalized‐ensemble simulation system (GENESIS) (Jung et al., 2024) predicted that neither the IDR nor the NTS alone formed condensates, whereas NTS‐IDR fragments clustered into two dense droplets (see Materials and Methods for details; Figure 6C). Consistently, only NTS‐IDR fused eGFP (NTS‐IDR‐eGFP) formed discrete nuclear puncta in Arabidopsis protoplasts (Figure 6B, lower panel). Together, these results demonstrate that the cooperative action of the NTS and IDR drives WSS1A LLPS.

### WSS1A–SUMO3 interaction modulates leaf senescence

**Figure 7:**
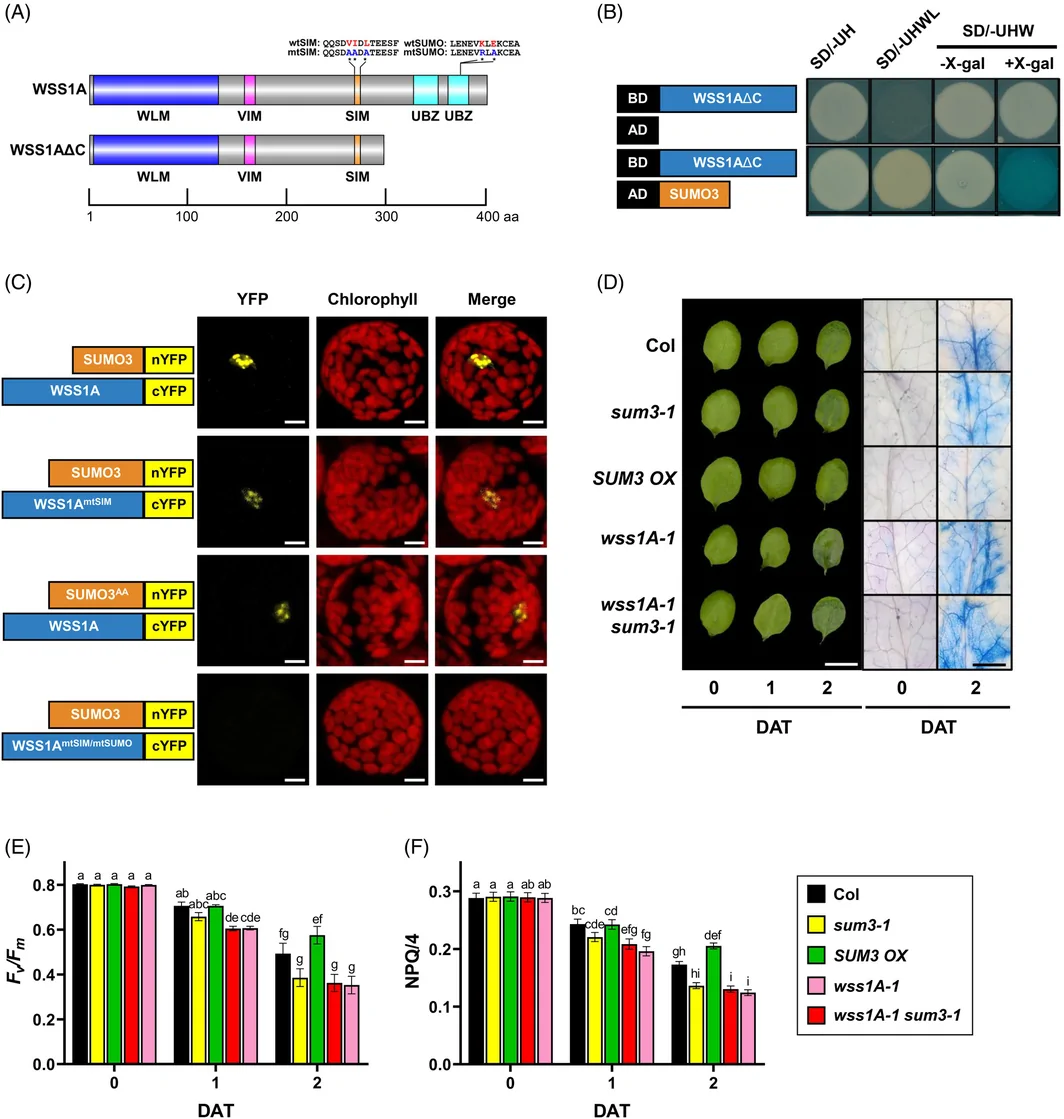
Protein–protein and genetic interactions between WSS1A and SUMO3. (A) Schematic representation of the full‐length and C‐terminal truncated form (WSS1AΔC) of WSS1A proteins with predicted SUMO‐interacting motif (SIM) and SUMOylation site. wtSIM and mtSIM, wild‐type and mutated SIM sequences; wtSUMO and mtSUMO, wild‐type and mutated SUMOylation site sequences. (B) WSS1A and SUMO3 protein–protein interaction tested in yeast. Yeast cells expressing WSS1AΔC fused with GAL4 DNA‐binding domain (BD) and SUMO3 fused with B42 activation domain (AD) were plated on selective media (SD/‐UHWL or SD/‐UHW with X‐gal) for reporter gene activation. ‐UHWL, without uracil, histidine, tryptophan, and leucine; X‐gal, 5‐Bromo‐4‐chloro‐3‐indolyl‐β‐D‐galactoside. (C) Bimolecular fluorescence complementation (BiFC) assays showing YFP fluorescence in Arabidopsis protoplasts co‐expressing SUMO3 variants (SUMO3 or SUMO3^AA^) fused to nYFP and WSS1A variants (WSS1A, WSS1A^mtSIM^, or WSS1A^mtSIM/mtSUMO^) fused to cYFP. SUMO3^AA^, a mutant version of SUMO3 where the di‐glycine (GG) motif was mutated to di‐alanine (AA), thereby preventing SUMOylation. Yellow, YFP signal; Red, autofluorescence for chlorophyll. Scale bar, 10 μm. (D–F) Leaf senescence phenotypes of Col, *sum3‐1*, *sum3 OX*, *wss1A‐1*, and *wss1A‐1 sum3‐1* leaves upon 30 μM cis‐platin (cis‐Pt) treatment at 0, 1, and 2 DAT. Representative images (D, left), TB dye staining (D, right), *F*_v_/*F*_m_ (E), and NPQ/4 (F) are shown. Data are presented as mean ± SEM (*n* = 8). Relative expression differences were compared using a two‐way anova followed by Tukey's multiple‐comparison test; different letters indicate statistically significant differences between groups. All experiments were performed with at least three independent biological replicates, and representative results are shown. Scale bar, 1 cm in (D).

To further explore the molecular mechanisms governing WSS1A function in leaf senescence, we conducted a yeast‐two‐hybrid (Y2H) screening with a truncated form of WSS1A (WSS1AΔC; amino acids 1–299) as bait and identified SUMO3 as a potential interacting partner (Figure 7A,B). This interaction was confirmed *in planta* by bimolecular fluorescence complementation (BiFC), which revealed YFP signals in the nucleus, including the nuclear puncta (Figure 7C; Figure S9).

Since web‐based prediction algorithms (see Materials and Methods) identified both a potential SUMOylation site and a SIM within WSS1A (Figure 7A), we questioned whether WSS1A undergoes SUMOylation or interacts with SUMOylated proteins through a SIM. To distinguish between covalent SUMOylation and non‐covalent SIM–SUMO interactions, we first generated a SIM‐mutated WSS1A (WSS1A^mtSIM^) and tested its interaction with SUMO3. BiFC analysis showed that WSS1A^mtSIM^ retained interaction with SUMO3 (Figure 7C; Figure S9), suggesting the possibility that WSS1A is SUMOylated via SUMO3 in the absence of an intact SIM, or that WSS1A contains an additional, non‐canonical SIM that mediates the interaction.

Furthermore, a SUMOylation‐defective SUMO3 mutant (SUMO3^AA^) could still interact with WSS1A, indicating a non‐covalent interaction with SUMO3. It was noticeable that the interactions between WSS1A^mtSIM^ and SUMO3, as well as WSS1A and SUMO3^AA^, exhibited slightly reduced YFP signal intensity and a lower frequency of fluorescent puncta. By contrast, mutation of both the SIM and the SUMOylation site (WSS1A^mtSIM/mtSUMO^) abolished the interaction (Figure 7C). Nonetheless, these results imply that WSS1A may participate both in SUMOylation via its SUMOylation site and in non‐covalent interaction with SUMOylated proteins through its SIM.

Given that SUMO3 interacted with WSS1A, we next aimed to investigate whether SUMO3 contributes to regulating cis‐Pt‐induced leaf senescence. Upon treatment with 30 μM cis‐Pt, leaves of a loss‐of‐function mutant of *SUMO3* (*sum3‐1*) exhibited accelerated senescence and chlorosis compared with Col leaves, with a significant reduction in photochemical efficiency and NPQ, as well as an increase in cell death, whereas *SUMO3*‐overexpressing (*SUM3 OX*) leaves exhibited more delayed senescence phenotypes (Figure 7D–F). Notably, the *wss1A‐1 sum3‐1* mutant exhibited a senescence phenotype similar to *wss1A‐1*, with no further enhancement of leaf senescence (Figure 7D–F), indicating that WSS1A and SUMO3 function in the same pathway to cooperatively regulate cis‐Pt‐induced leaf senescence. Collectively, these findings define a WSS1A–SUMO3 functional module in which SUMO3‐dependent regulation and LLPS of WSS1A modulate leaf senescence in Arabidopsis.

**Figure 8:**
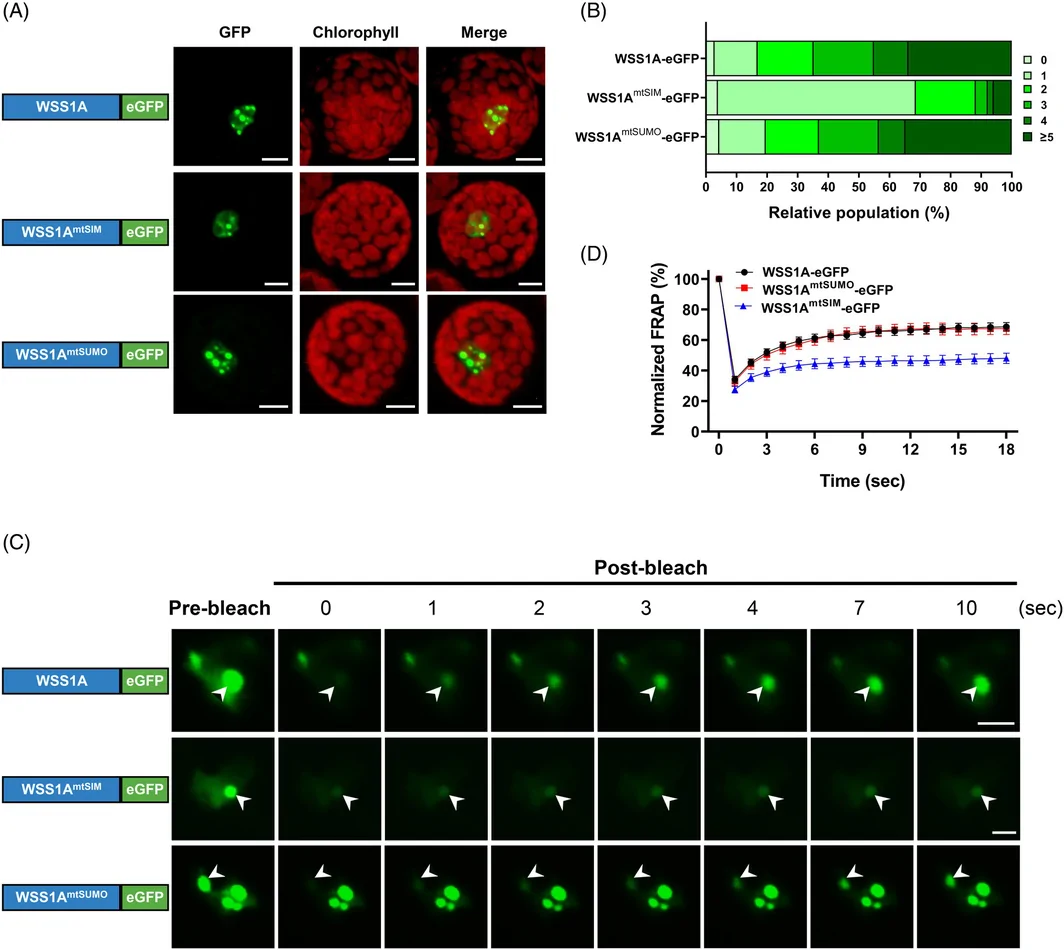
The functional role of the SUMO‐interacting motif (SIM) in WSS1A condensate formation and dynamics. (A) Representative confocal microscopy images of Arabidopsis mesophyll protoplasts with transiently expressing WSS1A variants (WSS1A, WSS1A^mtSIM^, and WSS1A^mtSUMO^) fused to eGFP. Green, GFP signal; Red, autofluorescence for chlorophyll. Scale bar, 10 μm. (B) Relative distribution of nuclear puncta number in Arabidopsis mesophyll protoplasts transiently expressing WSS1A variants shown in (A). The number of puncta was counted from 54, 51, and 55 nuclei observed in three independent experiments for WSS1A‐eGFP, WSS1A^mtSIM^‐eGFP, and WSS1A^mtSUMO^‐eGFP, respectively. (C) Representative images of nuclear condensates in WSS1A‐eGFP, WSS1A^mtSIM^‐eGFP, and WSS1A^mtSUMO^‐eGFP in a mesophyll protoplast analyzed by fluorescence recovery after photobleaching (FRAP) experiments. The bleaching laser intensity was 100%, and the excitation wavelength was 488 nm. White arrowheads indicate photo‐bleached WSS1A‐eGFP condensates. Scale bar, 10 μm. (D) FRAP recovery plot of nuclear condensates in (C). The graph represents mean ± SEM (*n* = 10). FRAP experiments were performed with at least three independent biological replicates, and representative results are shown.

SUMOylation has emerged as an important regulator of nuclear condensates, providing both scaffold‐like and solubilizing effects through multivalent SUMO–SIM interactions (Keiten‐Schmitz et al., 2021). Such interactions can promote the recruitment of client proteins into phase‐separated compartments (Alghoul et al., 2023; Ren et al., 2024). Because WSS1A interacts with SUMO3 and contains both a putative SIM and a SUMOylation site, we asked whether these motifs contribute to the condensate behavior of WSS1A (Figure 8A). Fluorescence microscopy analysis revealed that WSS1A^mtSIM^‐eGFP exhibited a marked reduction in the number of nuclear puncta, whereas WSS1A^mtSUMO^‐eGFP formed nuclear condensates at levels comparable with wild‐type WSS1A‐eGFP (Figure 8B). Consistently, in FRAP experiments, WSS1A^mtSIM^‐eGFP droplets displayed a significantly reduced recovery plateau (0.47), reflecting decreased molecular mobility. By contrast, WSS1A^mtSUMO^‐eGFP retained a recovery plateau (0.72), comparable to that of WSS1A‐eGFP (Figure 8C,D). Together, these findings suggest that the SIM, rather than the predicted SUMOylation site, is crucial for the condensate‐forming property of WSS1A. Furthermore, this differential requirement for the SIM domain demonstrates that WSS1A condensation is driven by specific molecular interactions rather than a non‐specific artifact of protein concentration, which may be requisite for the establishment of functional repair centers.

## DISCUSSION

Accumulating evidence suggests that DPCs play vital roles in genomic instability across diverse species, including humans, where they compromise cellular function and ultimately trigger premature aging (Lessel et al., 2014; Ming et al., 2020). However, the impact of DPC accumulation and repair on plant senescence has remained largely unresolved. In this study, we demonstrate that cis‐Pt, a potent DPC inducer, promotes leaf senescence in Arabidopsis (Figure 3), revealing a previously unrecognized link between genotoxic stress by cis‐Pt and plant senescence. We further establish WSS1A as an evolutionarily conserved DPC repair protease that functions as a key negative regulator of leaf senescence in Arabidopsis (Figures 1, 2, and 4; Figure S4). Beyond its physiological role, WSS1A undergoes LLPS within the nucleus through the cooperative action of its NTS and IDR and shows genetic and molecular interaction with SUMO3 (Figures 5, 6, 7, 8). Taken together, these findings support a model in which DPC repair, SUMO3‐mediated regulation, and the condensate‐forming properties of WSS1A are integrated to modulate leaf senescence in Arabidopsis.

Leaf senescence is characterized by coordinated physiological and molecular changes, including the decline of photosynthetic activity and extensive transcriptional reprogramming (Guo & Gan, 2012; Lim et al., 2007; Woo et al., 2019). In our study, cis‐Pt treatment faithfully recapitulated these hallmarks (Figure 3; Figure S6), suggesting a potential connectivity where DPC accumulation can act as an upstream trigger that engages endogenous senescence regulatory programs. Because DPCs physically obstruct essential DNA‐dependent processes, including transcription and replication, their progressive accumulation leads to severe transcriptional stalling and replication stress (Ji et al., 2018; Permana & Snapka, 1994). In this context, we propose that WSS1A functions as a primary proteolytic safeguard, by efficiently resolving these genotoxic lesions; consequently, our findings highlight that WSS1A‐mediated DPC repair is a critical molecular mechanism required to maintain genome integrity and transcriptional homeostasis under senescence‐inducing conditions (summarized in the schematic model; Figure S11).

Defects in the mammalian DPC repair protease SPRTN are associated with shortened lifespan and premature aging phenotypes (Lessel et al., 2014), but the contribution of DPC repair pathways to plant senescence has yet to be elucidated. Building on this framework, we investigated the functional relevance of WSS1A in leaf senescence. Under both cis‐Pt‐induced genotoxic stress, as well as during age‐dependent and dark‐induced senescence, WSS1A consistently acted as a negative regulator of leaf senescence (Figures 2 and 4; Figure S4), supporting its broad role in suppressing leaf senescence under diverse conditions. These observations suggest that WSS1A contributes to the maintenance of leaf longevity, likely through preserving genome integrity, and point to a conserved role of DPC repair mechanisms in regulating aging across eukaryotic kingdoms. Although this study focuses on WSS1A, DPC repair is mediated by multiple, partially overlapping pathways in plants. In our initial analysis of DPC repair‐related mutants—including *ddi1‐2*, *mus81‐1*, *tdp1‐2*, *wss1A‐1*, and *wss1B‐1*—*wss1A‐1* displayed the most prominent premature senescence phenotype under dark‐induced senescence conditions (Figure S1). This suggests that WSS1A may represent a particularly crucial DPC repair factor in the regulation of leaf senescence. However, the milder phenotypes of the other mutants do not exclude their potential contribution to specific types of DPC repair or to senescence under different stress conditions. Future genetic and biochemical analyses combining these repair factors with WSS1A will be required to determine how distinct DPC repair pathways are coordinated during leaf senescence.

DPC repair‐related metalloproteases (containing either a WLM or Spr‐T domain) are evolutionarily classified into three major branches: the Wss1 branch, the N‐terminal ubiquitin‐like (UBL)‐Wss1 branch, and the Spr‐T/SPRTN branch (Stingele et al., 2015). The Wss1 branch includes canonical proteases such as yeast Wss1 and Arabidopsis WSS1A, while the UBL‐Wss1 proteins, including Arabidopsis WSS1B, contain an additional UBL domain. The SPRTN branch has diverged substantially in both structure and function. Notably, our cross‐kingdom complementation assays confirmed that WSS1A, but not WSS1B, can rescue DPC repair defects in yeast *wss1* mutants (Figure 1D,E). Similarly, Arabidopsis *wss1A* knockout mutants exhibit retarded seedling growth upon CPT or cis‐Pt treatments, unlike the *wss1B* knockout mutants (Enderle et al., 2019). Together, these findings highlight the functional divergence among the WLM protease subfamilies and establish WSS1A as a conserved functional ortholog of yeast Wss1. Interestingly, WSS1A also possesses two UBZ domains, a feature shared with mammalian SPRTNs (Enderle et al., 2019; Stingele et al., 2015; Figure 1A). Since the UBZ domain in SPRTNs preferentially interacts with ubiquitin (Zhao et al., 2021), it is plausible that WSS1A may facilitate interactions with ubiquitinated substrates or mediate proteasome‐coupled DPC repair. Further exploration of WSS1A as an integrator of diverse post‐translational modification (PTM) signals will shed light on our understanding of proteolytic DPC repair mechanisms in plants.

DPC repair is tightly regulated by PTMs such as SUMOylation, ubiquitination, and PARylation in various organisms (Borgermann et al., 2019; Essawy et al., 2023; Fábián et al., 2024; Ruggiano et al., 2021; Sun et al., 2021). These PTMs not only serve as damage‐recognition signals but also orchestrate the recruitment and activation of DPC repair proteases. In mammals, ubiquitin recruits SPRTN to DPC sites while simultaneously regulating its autocatalytic cleavage, thus ensuring controlled proteolytic activity (Zhao et al., 2021). In yeast, Wss1 is recruited to SUMOylated DPCs via interactions with Cdc48 and DEGRADATION OF ALPHA 1 (Doa1), which forms a SUMO‐dependent proteolytic complex that resolves lesions inaccessible to conventional repair pathways (Balakirev et al., 2015). By contrast, the role of PTMs in plant DPC repair has remained poorly understood. In this study, we found that WSS1A associates with SUMO3 via its SIM and is also subject to covalent SUMOylation (Figure 7C; Figure S9), suggesting multilayered SUMO‐dependent regulation. These modifications may modulate WSS1A activity, stability, or interaction networks, potentially linking DPC repair to proteasome‐mediated degradation pathways. Elucidating how SUMOylation interfaces with other PTMs, including ubiquitination and PARylation, will be essential for understanding the regulatory architecture of plant DPC repair.

The functional relevance of SUMO3 in leaf senescence is further underscored by its role in cis‐Pt‐induced leaf senescence. *sum3‐1* exhibited accelerated senescence, whereas *SUM3 OX* plants delayed senescence under cis‐Pt‐induced genotoxic stress (Figure 7D–F). Notably, the *wss1A‐1 sum3‐1* double mutant did not exhibit an enhanced senescence phenotype relative to the *wss1A‐1* single mutant, suggesting that WSS1A and SUMO3 function within the same genetic pathway. This epistatic relationship supports a model in which SUMO3 acts upstream of, or in concert with, WSS1A to facilitate DPC repair and attenuate cis‐Pt‐induced leaf senescence.

At the mechanistic level, we discovered that WSS1A undergoes LLPS to form nuclear condensates primarily driven by its NTS and IDR (Figures 5 and 6), implying its potential role in the formation of repair condensates at DPC sites. Emerging evidence suggests that LLPS plays a role in DPC repair. For example, the mammalian DPC protease SPRTN exhibits liquid‐like properties within nuclear speckles (Stingele et al., 2016), although further studies are required to determine whether they truly undergo LLPS, and the scaffold protein SLX4 forms phase‐separated condensates to compartmentalize SUMO‐dependent repair machinery at DNA lesions (Alghoul et al., 2023). We propose that the formation of WSS1A nuclear condensates represents a sophisticated spatial strategy to increase the local concentration of the repair machinery at DPC lesions. By compartmentalizing DPC repair factors within specific chromatin environments—such as the heterochromatic chromocenters—these LLPS‐like structures potentially facilitate efficient genome maintenance. Consequently, this specialized subnuclear targeting safeguards cells against premature leaf senescence under both natural and cis‐Pt‐induced genotoxic stress conditions. Consistent with this notion, we further demonstrated that non‐covalent interaction with SUMO3 via the SIM is a key driver of WSS1A LLPS. Disruption of the SIM substantially reduced both the formation and fluidity of WSS1A condensates, whereas disruption of its SUMOylation site had minimal impact (Figure 8). A similar observation has been reported in the mammalian DNA repair‐associated scaffold protein, SLX4, where SUMO–SIM interactions promote the assembly of phase‐separated repair hubs at sites of DNA damage (Alghoul et al., 2023). These consistent observations imply that SUMO–SIM interactions are broadly required for the proper assembly and localization of phase‐separated repair factors. Furthermore, studies on SUMO‐dependent recruitment of WSS1A to DPC sites will enhance our understanding of the functional significance of WSS1A phase separation in genome maintenance and developmental processes, including leaf senescence.

Finally, while our study establishes the importance of WSS1A in controlling leaf senescence, the specific identity of senescence‐relevant DPC substrates remains to be fully characterized. In mammals, well‐defined DPC substrates include RNA polymerase II, topoisomerases, and histone‐modifying enzymes, many of which are also implicated in aging‐related pathways (Larsen et al., 2019; Weickert et al., 2023). Identifying plant DPC‐associated proteins using liquid chromatography–tandem mass spectrometry (LC–MS/MS)‐based proteomics and determining whether WSS1A condensates co‐localize with specific chromosomal regions or canonical DNA damage markers will be the critical next steps. Such analyses will further clarify how spatially organized DPC repair contributes to genome maintenance and leaf longevity.

## MATERIALS AND METHODS

### Plant materials and growth conditions

*Arabidopsis thaliana* plants in the Col ecotype background were used as wild‐type in this study. All plant materials were grown in a controlled growth room at 22°C under 16‐h light/8‐h dark photoperiod. *ddi1‐2* (SAIL_1239_C12; Lin et al., 2011), *ore1‐2* (Kim et al., 2009), *nore1‐1* (Lee et al., 2016), *mus81‐1* (GABI_113F11; Hartung et al., 2006), *sum3‐1* (SM_3_2707; Ingole et al., 2021), *tdp1‐2* (SALK_119060; Enderle et al., 2019), *wss1A‐1*, and *wss1B‐1* (CRISPR‐Cas9‐mediated loss‐of‐function mutants; Enderle et al., 2019) have been previously described. The T‐DNA insertion site of *wss1A‐4D* (SAIL_815_C01, a gain‐of‐function mutant) obtained from the SAIL collection was verified by PCR‐based genotyping, and the transcript level of *WSS1A* was confirmed by reverse transcription‐quantitative polymerase chain reaction (RT‐qPCR) (Figure S3). To generate transgenic Arabidopsis plants overexpressing *WSS1A* or *SUMO3* (Figure S10), the coding sequence (CDS) of *WSS1A* and *SUMO3* was amplified by RT‐PCR, subcloned into the *pCR‐CCD‐F* vector, and then recombined into the *pCsVMV* Gateway binary vector, which contains the *CsVMV* promoter and an N‐terminal HA or GFP tag using Gateway™ LR clonase™ II Enzyme mix (Invitrogen™, Carlsbad, USA). Primers used for genotyping and vector construction are listed in Table S1.

### Leaf senescence assays

All leaf senescence experiments were conducted using the third and fourth rosette leaves. For the dark‐induced leaf senescence assay, leaves detached at 12 DAE were floated in a 3 mM 2‐(N‐morpholino)ethanesulfonic acid (MES) buffer (pH 5.8) and then incubated in the darkness at 22°C for the indicated number of days. The age‐dependent senescence assay was performed using leaves detached from plants at 8‐day intervals between 12 and 28 DAE. To perform the cis‐Pt‐induced senescence assay, leaves detached at 12 DAE were floated in a 3 mM MES buffer containing either 15 or 30 μM cis‐Pt (Invitrogen™, Carlsbad, USA). Leaf senescence‐related parameters, including total chlorophyll contents, photochemical efficiency, NPQ/4, membrane ion leakage, and cell death (as assessed by TB staining), were evaluated as previously described (Kim et al., 2009; Kim et al., 2018; Ritchie, 2006; Woo et al., 2001).

### Yeast complementation and two‐hybrid assays

For yeast complementation assays, the yeast strains for *Tdp1* and/or *Wss1* deletion (*Δtdp1*, *Δwss1*, and *Δtdp1 Δwss1*) and *p415‐pADH:Wss1* were obtained from the previous research (Stingele et al., 2014). Arabidopsis *WSS1A* and *WSS1B* were cloned into *p415‐pADH* plasmid (*pRS415*), transformed into yeast (Mumberg et al., 1995), and selected on synthetic dropout (SD) medium lacking leucine (SD/‐L). To check CPT sensitivity, exponentially grown cells were serially diluted, spotted onto SD/‐L agar plates supplemented with 40 μM CPT (Invitrogen™, Carlsbad, USA), and incubated for 2.5 days at 30°C. To evaluate formaldehyde sensitivity, cells were pre‐treated with 40 mM formaldehyde for 15 min, washed twice with phosphate‐buffered saline, spotted onto SD/‐L plates, and incubated for 2.5 days at 30°C.

Y2H screening was performed using the AH109 strain carrying the *pGBKT7: WSS1AΔC* construct which contains a CDS fragment corresponding to amino acids 1–299 of WSS1A to prevent transcriptional autoactivation, as bait. A total of 3.60 × 10^6^ colonies from an Arabidopsis vegetative tissue cDNA library (Clontech Laboratories, Mountain View, USA) were screened. To examine the protein–protein interaction between WSS1A and SUMO3, the *WSS1AΔC* was fused to the GAL4 DNA‐binding domain in the *gGilda* plasmid (Gateway version of *pGilda*), and full‐length *SUMO3* CDS or *SUMO3* ^*AA*^ CDS was cloned into the GAL4‐DNA activation domain plasmid *gOST4‐5* (Gateway version of *pOST4‐5*). The mutated constructs (e.g., WSS1A^mtSIM^, WSS1A^mtSIM/mtSUMO^, and SUMO3^AA^) were generated following the QuickChange site‐directed mutagenesis protocol (Agilent Technologies, Santa Clara, USA), with PCR amplification using High‐Fidelity PCR Master Mix (New England Biolabs, Ipswich USA). These constructs were co‐transformed into the yeast YM4271 cells following the previously described manual (Gietz & Schiestl, 2007), which were grown on SD/‐UH (medium without uracil and histidine), SD/‐UHWL (medium without uracil, histidine, tryptophan, and leucine), and SD/‐UHW supplemented with 5‐bromo‐4‐chloro‐3‐indolyl‐β‐D‐galactopyranoside (X‐gal) or without X‐gal. Plates were incubated at 30°C for 2–6 days. PCR primers are listed in Table S1.

### RNA extraction and gene expression analysis

Total RNA was isolated from third and fourth rosette leaves of Arabidopsis plants using the WelPrep™ Total RNA isolation kit (Welgene, Gyeongsan, Republic of Korea), and cDNA was synthesized using PrimeScript™ RT Master Mix (Takara Bio, San Jose, USA). Quantitative PCR was performed using the TOPreal™ SYBR Green qPCR Premix (Enzynomics, Daejeon, Republic of Korea) in a CFX96 Touch Real‐Time PCR detection system (Bio‐Rad, California, USA) according to the manufacturer's instructions. Three independent biological replicates were analyzed, and the relative expression level of each gene was normalized to that of *ACTIN2* (*ACT2*). PCR primers are listed in Table S1.

### mRNA‐seq and transcriptomic analysis

Leaves detached from Col plants at 12 DAE were floated on 3 mM MES (pH 5.8) with or without 30 μM cis‐Pt and incubated for 1 day. The mRNA‐seq experiments were performed in two independent biological replicates per treatment (0 and 30 μM cis‐Pt). Total RNA was isolated from the leaves using QIAzol (QIAGEN, Venlo, Germany), and its integrity was assessed using Agilent Technologies 2100 Bioanalyzer (Agilent Technologies, Santa Clara, USA). Total RNA samples with an RNA integrity number (RIN) greater than 7.8 were used for mRNA‐seq analysis. Poly(A) mRNA was isolated from the total RNA (2 μg) and fragmented using Illumina TruSeq™ Stranded mRNA LT Sample Prep Kit (Illumina, San Diego, USA) with poly‐T oligo‐attached magnetic beads. After the cDNA synthesis from the RNA fragments using Superscript II reverse transcriptase (Invitrogen™, Carlsbad, USA), strand‐specific cDNA libraries were generated by adaptor ligation and sequenced by Illumina NovaSeq 6000 System (Illumina, San Diego, USA), according to the Illumina Directional RNA‐Seq protocol.

To analyze RNA‐seq data, TruSeq universal and indexed adapter sequences were trimmed from the raw reads using Cutadapt (v1.12) and the cleaned reads were then aligned to the Arabidopsis reference genome (TAIR10; Lamesch et al., 2012) using STAR (v2.7.10a) (https://github.com/alexdobin/STAR) with default settings. Mapped reads were subsequently counted for gene features (TAIR10) using HTSeq (v0.9.1). Genes with counts per million (CPM) values greater than 1 in at least one sample were retained. A pseudo‐count of 1 was added to all CPM values, and the resulting CPM + 1 values were log_2_‐transformed. A two‐tailed test was used to compute adjusted *P*‐values for the observed *t*‐value and log_2_‐median ratio of each gene. The *P*‐values were combined using Stouffer's method (Hwang et al., 2005) to generate an overall *P*‐value. Genes were considered DEGs if the overall *P*‐value was <0.05 and the absolute log_2_(FC) was greater than 1.0 a threshold representing the mean of the 5th and 95th percentiles of the empirical null distribution.

To identify cellular processes significantly enriched among the DEGs, functional enrichment analysis was conducted using Metascape (https://metascape.org/, assessed on April 16, 2025) (Zhou et al., 2019). The analysis was performed with default parameters, including GO biological processes, a significance threshold of *P*‐value <0.01, a minimum gene count of three, and an enrichment factor greater than 1.0 (defined as the ratio between the observed counts and the expected counts by random chance).

### *In silico* analyses

Putative SIM and SUMOylation site in WSS1A were identified through computational tools (GPS‐SUMO [http://sumosp.biocuckoo.org/online.php], SUMOplot [http://www.abgent.com/sumoplot], and JASSA [http://www.jassa.fr/index.php?m=jassa]). Phase separation propensity was calculated using three prediction tools: MolPhase, DeePhase, and FuzDrop, which evaluate the likelihood of phase‐separated biomolecular condensates (Hardenberg et al., 2020; Liang et al., 2024; Saar et al., 2021). The putative IDR in WSS1A was predicted using AIUPred and AlphaFold 3 (Abramson et al., 2024; Erdős & Dosztányi, 2024). The NTS in WSS1A was characterized by using CIDER and ProtScale to evaluate net charge per residue and hydrophilicity (Holehouse et al., 2017; Wilkins et al., 1999).

### Microscopic analyses

Subcellular localization analyses were performed following the standard protocols. In brief, the full‐length CDS of *WSS1A, WSS1A* ^*mtSIM*^, and *WSS1A* ^*mtSUMO*^ and sequences of *NTS*, *IDR*, *NTS*‐fused *IDR* in *WSS1A* were individually inserted into a plant expression vector (gateway version of *pCsV999‐eGFP*) using Gateway™ LR clonase™ II Enzyme mix (Invitrogen™, Carlsbad, USA). The constructs were transfected into Arabidopsis mesophyll protoplasts (Yoo et al., 2007). To study the subcellular localization of WSS1A in *Nicotiana benthamiana* leaves, full‐length CDS of *WSS1A* was cloned into the *pCsVMV* Gateway binary vector under the control of the *CsVMV* promoter and an N‐terminal eGFP tag. The WSS1A‐GFP was expressed in *N. benthamiana* by *Agrobacterium tumefaciens*‐mediated transient expression assays (Wydro et al., 2006).

For BiFC analyses, the full‐length CDS of *WSS1A*, *WSS1A* ^*mtSIM*^, *WSS1A* ^*mtSIM/mtSUMO*^, *SUMO3*, and *SUMO3* ^*AA*^ were individually amplified and recombined into both plant expression vectors (gateway version of *pSPYNE* or *pSPYCE*) using Gateway™ LR clonase™ II Enzyme mix (Invitrogen™, Carlsbad, USA). The YFP fragment‐fused constructs were co‐transformed into Arabidopsis mesophyll protoplasts. PCR primers are listed in Table S1.

### FRAP analyses

FRAP analyses were performed *in vivo* using a 40× oil immersion objective of the FV1200 Olympus confocal microscope (Olympus, Tokyo, Japan). The photobleaching laser was set to 100%, and the excitation wavelength was 488 nm. The GFP fluorescence recovery was recorded every 1 sec. The Fiji image processing software was used to obtain images and perform fluorescence intensity analysis.

### Expression and purification of recombinant WSS1A proteins and *in vitro* phase separation assays

To analyze *in vitro* LLPS, we constructed expression vectors encoding six consecutive histidine residues (His6‐tag) with the MBP tag, into which the WSS1A‐eGFP or eGFP was individually inserted using T4 ligase (New England Biolabs, Ipswich, USA). Each construct was transformed into the *Escherichia coli* Rosetta (DE3) strain. More than 1 L of each bacterium was induced to express proteins with 0.5 mM isopropyl‐β‐D‐thiogalactopyranoside (IPTG), followed by a collection of cells with centrifugation (5000 ***g***, 30 min). Then the cells were lysed with native lysis buffer (50 mM NaH_2_PO_4_, 500 mM NaCl, pH 8.0) and sonicated using an ultrasound method for 30 min. Subsequently, the lysates were centrifuged (12 000 ***g***, 30 min) to pellet the cellular debris, and the supernatant was retained and then purified with His60 Ni Gravity Column Purification Kit (Takara Bio, San Jose, USA). His‐tagged target proteins were confirmed using standard SDS‐PAGE and Coomassie staining methods.

The purified His‐tagged recombinant proteins were diluted with elution buffer (50 mM NaH_2_PO_4_, 300 mM NaCl, 300 mM imidazole, pH 7.4), to the appropriate concentrations. To induce phase separation, different concentrations of target proteins were incubated for 15 min at room temperature with and without PEG 8000 at a final concentration of 10% (w/v). After incubation, the turbidity of the fusion protein solution was measured (optical density at 600 nm). To visualize the phase separation, droplets were monitored with Zeiss LSM 7 DUO Confocal Microscope (Zeiss, Oberkochen, Germany).

### Coarse‐grained models for NTS and IDR of WSS1A

A coarse‐grained (CG) simulation approach was employed to model WSS1A proteins using GENESIS (Jung et al., 2024). A bead‐based CG model was constructed, with each residue represented by a particle centered on its Cα atom. Molecules were duplicated into a 2 × 2 × 30 grid, and a shrinking simulation adjusted the periodic boundary to a target volume of 200 Å × 200 Å × 4000 Å. This was followed by a 100 nanosecond production run with implicit solvent and constant temperature (300 K). Hydrophobicity parameters were taken from Regy et al. (2021), and simulations were subsequently visualized using VMD (Humphrey et al., 1996).

### Statistical analysis

For statistical analysis, GraphPad Prism 8 was used. For all graphs, data were represented as SEM. All details of the statistics applied are provided in the figure legend.

## AUTHOR CONTRIBUTIONS

SP and HRW contributed to the conceptualization of the study. SP, HO, UJ, HC, HP, YK, JK, and YSY performed experiments. SP, JHL, and JK analyzed the data. SP and HRW wrote the original draft of the manuscript. ZL and J‐CL contributed to reviewing and editing the manuscript.

## CONFLICT OF INTEREST

The authors declare no competing interests.

## Supporting information


**Figure S1.** Leaf senescence phenotypes of loss‐of‐function mutants of five key components in the DPC repair pathway in Arabidopsis.
**Figure S2.** Sequence conservation and structural similarity of the WLM domain in WSS1A, WSS1B, and Wss1.
**Figure S3.** Analysis of the *wss1A‐4D* mutant.
**Figure S4.** Age‐dependent leaf senescence phenotypes of *wss1A‐1* and *wss1A‐4D*.
**Figure S5.** Leaf senescence phenotypes induced by various DPC‐inducing agents.
**Figure S6.** cis‐Pt‐induced leaf senescence phenotypes of Col.
**Figure S7.** Subcellular localization of the WSS1A‐eGFP fusion protein in Arabidopsis.
**Figure S8.** FoldIndex analysis of WSS1A.
**Figure S9.** Protein–protein interactions between WSS1A variants and SUMO3 variants.
**Figure S10.** Expression analysis of the *WSS1A* and *SUMO3 OX* transgenic plant.
**Figure S11.** A proposed working model for WSS1A‐mediated DPC‐proteolysis repair in the regulation of leaf senescence in Arabidopsis.
**Table S1.** Primers used in this work.

**Data S1.** Differentially expressed genes by cis‐Pt treatment and/or along leaf age.

## Data Availability

The mRNA‐seq data generated in this study have been deposited in Sequence Read Archive (SRA) in National Genomics Data Center under accession number PRJNA1251531 and Korean Sequence Read Archive (KRA) under accession number KAP241507.
