# Supplementary material for: Arabidopsis WSS1A, a DNA‐protein crosslink repair protease, delays leaf senescence in cooperation with SUMO3
Source: Plant J. 2026 Aug 22;127(4):e71094. doi: 10.1111/tpj.71094 (PMC13499631; doi:10.1111/tpj.71094)
Supplement: Supplementary file 1 [file TPJ-127-0-s001.docx]

**Arabidopsis WSS1A, a DNA-Protein Crosslink Repair Protease, Delays Leaf Senescence in Cooperation with SUMO3**

Sanghoon Park^1^, Hyunwoo Oh^1^, Ukcheol Jeong^1^, Jae Ho Lee^2^, Hyeyoung Choi^1^, Hyunsu Park^1^, Jinkwang Kim^1^, Yongmin Kim^1,3^, Junmin Kwak^1^, Yeong Seon Yoon^1^, Zhonghai Li^4^, Jong-Chan Lee^1,5^, Hye Ryun Woo^1,5,*^

^1^Department of New Biology, Daegu Gyeongbuk Institute of Science and Technology (DGIST), Daegu, 42988, Korea

^2^Korean Bioinformation Center (KOBIC), Korea Research Institute of Bioscience and Biotechnology (KRIBB), Daejeon, 34141, Korea

^3^Baekdudaegan Global Seed Vault (BGSV), Korea Arboreta and Gardens Institute (KoAGI), Bonghwa-gun, 36209, Korea

^4^State Key Laboratory of Tree Genetics and Breeding, College of Biological Sciences and Technology, Beijing Forestry University, Beijing, 100083, China

^5^New Biology Research Center, Daegu Gyeongbuk Institute of Science and Technology (DGIST), Daegu, 42988, Korea

^*^Correspondence: Hye Ryun Woo, hrwoo@dgist.ac.kr

**Supporting information**

**Figure S1. Leaf senescence phenotypes of loss-of-function mutants of 5 key components in the DPC repair pathway.**

**Figure S2. Sequence conservation and structural similarity of the WLM domain in WSS1A, WSS1B, and Wss1.**

**Figure S3. Analysis of the *wss1A-4D* mutant.**

**Figure S4. Age-dependent leaf senescence phenotypes of *wss1A-1* and *wss1A-4D.***

**Figure S5. Leaf senescence phenotypes induced by various DPC-inducing agents.**

**Figure S6. cis-Pt-induced leaf senescence phenotypes of Col.**

**Figure S7. Subcellular localization of the WSS1A-eGFP fusion protein in Arabidopsis.**

**Figure S8. FoldIndex analysis of WSS1A.**

**Figure S9. Protein-protein interactions between WSS1A variants and SUMO3 variants.**

**Figure S10. Expression analysis of the WSS1A and SUMO3 OX transgenic plant.**

**Figure S11. A proposed working model for WSS1A-mediated DPC-proteolysis repair in the regulation of leaf senescence in *Arabidopsis*.**

**Table S1. Primers used in this study.**

**Data S1. Differentially expressed genes by cis-Pt treatment and/or along leaf age.**

**Figure S1. Leaf senescence phenotypes of loss-of-function mutants of 5 key components in the DPC repair pathway in Arabidopsis.**

**(a, b)** Representative image **(a)** and *F_v_/F_m_* **(b)** of Col, *ddi1-2*, *mus81-1*, *tdp1-2*, *wss1A-1*, and *wss1B-1* leaves during dark-induced senescence at 0, 3, 4, and 5 DAT. Data are presented as mean ± SEM (n = 8). Asterisks indicate significant differences (**p* < 0.05; ****p* < 0.001, two-way ANOVA followed by Dunnett’s multiple-comparison test).

**Figure S2. Sequence conservation and structural similarity of the WLM domain in WSS1A, WSS1B, and Wss1.**

**(a, b)** Multiple sequence alignment of the predicted WLM domains in Arabidopsis WSS1A **(a)** and WSS1B **(b)** with the yeast WLM domain, generated using Clustal Omega. The active site residues of WLM metalloprotease HExxH are marked by red circles. Asterisk (*), invariant residues; colon (:), highly similar residues; period (.), weak conservation between residues.

**(c)** Structural comparison of the WLM domains of WSS1A, WSS1B, and Wss1. Predicted WLM structure of WSS1A (green; Alphafold3) and WSS1B (orange; Alphafold3) were superimposed onto the crystal structure of the WLM domain of Wss1 (gray; PDB:5XBN), respectively.

**Figure S3. Analysis of the *wss1A-4D* mutant.**

**(a)** Schematic representation of the T-DNA insertion sites of the *wss1A-4D* mutant (SAIL_815_C01). The inverted triangle indicates the T-DNA insertion site in the 5’ untranslated region of *WSS1A*.

**(b)** Relative expression levels of *WSS1A* in *wss1A-4D* mutant compared with Col. *WSS1A* transcript levels were analyzed by RT-qPCR and normalized to *ACT2* transcript level. The values shown are relative to the level of Col. Data represent mean ± SEM (n = 3). The asterisk indicates a significant difference (****P* < 0.001, Student's *t*-test).

**Figure S4. Age-dependent leaf senescence phenotypes of *wss1A-1* and *wss1A-4D*.**

**(a-d)** Age-dependent leaf senescence phenotypes of the third and fourth leaves from Col, *wss1A-1*, and *wss1A-4D* plants at 12, 20, and 28 DAE. Representative images **(a)**, Changes in photochemical efficiency (*F_v_/F_m_*) **(b)**, NPQ/4 **(c)**, and chlorophyll content (mg/g FW) **(d)** are shown. Scale bar, 1 cm. Data are presented as mean ± SEM (n = 8). Asterisks indicate significant differences (**p* < 0.05; ***p* < 0.01; ****p* < 0.001, two-way ANOVA followed by Dunnett’s multiple-comparison test). All experiments were performed with at least three independent biological replicates, and representative results are shown.

**Figure S5. Leaf senescence phenotypes induced by various DPC-inducing agents.**

**(a, b)** Time-course analysis of leaf senescence phenotypes induced by 3 µM CPT, 15 µM cis-Pt, 20 µM etoposide, or 20 µM zebularine treatment in Col plants at 0 to 3 DAT. *F_v_/F_m_* **(a)** and NPQ **(b)** are shown. Data are presented as mean ± SEM (n = 8). Asterisks indicate significant differences (****p* < 0.001, two-way ANOVA followed by Dunnett’s multiple-comparison test). DAT, days after treatment.

**Figure S6. cis-Pt-induced leaf senescence phenotypes of Col.**

**(a, b)** Time-course analysis of leaf senescence phenotypes induced by 7.5 to 60 µM cis-Pt treatment in Col plants at 0 to 4 DAT. *F_v_/F_m_* **(a)** and NPQ **(b)** are shown. Data are presented as mean ± SEM (n = 8). Asterisks indicate significant differences; ***p* < 0.01; ****p* < 0.001, two-way ANOVA followed by Dunnett’s multiple-comparison test.

**Figure S7. Subcellular localization of the WSS1A-eGFP fusion protein** **in Arabidopsis.**

Confocal images of a root cell from a transgenic plant overexpressing WSS1A-eGFP. Note that WSS1A-eGFP puncta are preferentially localized within lightly DAPI-stained euchromatin regions but excluded from DAPI-dense chromocenters in the merged view. Green, GFP signal; Blue, DAPI staining (DNA). Scale bars, 10 µm.

**Figure S8**. **FoldIndex analysis of WSS1A.**

Predicted folded and unfolded regions of WSS1A based on FoldIndex analysis (Prilusky et al., 2005). Green, folded regions; Red, unfolded regions.

**Figure S9. Protein-protein interactions between WSS1A variants and SUMO3 variants.**

**(a)** BiFC assays showing no detectable YFP fluorescence in control combinations, including SUMO3 fused to nYFP with an empty insert fused to cYFP (upper panel) and WSS1A fused to cYFP with an empty insert fused to nYFP (lower panel).

**(b)** BiFC assays showing YFP fluorescence with WSS1A variants (wild-type or WSS1A^mtSIM^, the mutated SIM) fused to nYFP and SUMO3 variants (wild-type or SUMO3^AA^, the mutant blocking SUMOylation) fused to cYFP. Yellow, YFP signal; Red, autofluorescence for chlorophyll. Scale bar, 10 µm.

**Figure** **S10. Expression analysis of the *WSS1A* and *SUMO3 OX* transgenic plant.**

**(a, b)** Relative expression levels of *WSS1A* in the *WSS1A OX* (*gCsVMV*-*WSS1A-eGFP*) transgenic plant and **(a)** and *SUMO3* in the *SUM3 OX* (*gCsVMV*-*SUMO3-HA*) transgenic plant **(b)** compared with Col. *WSS1A* and *SUMO3* transcript levels were analyzed by RT-qPCR and normalized to *ACT2* transcript level. Expression values are presented relative to Col. Data represent mean ± SEM (n = 3). Asterisks indicate significant differences (****p* < 0.001, Student's *t*-test).

**Figure S11. A proposed working model for WSS1A-mediated DPC-proteolysis repair in the regulation of leaf senescence in *Arabidopsis*.**

**(Top)** Endogenous and exogenous sources of genotoxic stress, including increasing age, reactive oxygen species (ROS), and UV light, as well as the genotoxic agent cis-Pt, promote the formation of DPCs, in which cellular proteins become covalently trapped on genomic DNA (Erber et al., 2024; Hyang et al., 2020; Maskey et al., 2014; Stingele et al., 2015).

**(Bottom left)** In wild-type plants, WSS1A is recruited to DPC lesions and assembles into nuclear condensates through LLPS. Within these condensates, non-covalent SUMO3–SIM interactions promote WSS1A condensation and locally concentrate the repair machinery. WSS1A then proteolytically cleaves the crosslinked protein, removing the lesion and restoring intact DNA, thereby preserving genome stability and preventing premature leaf senescence.

**(Bottom right)** In loss-of-function mutants of *WSS1A*, DPCs remain unrepaired and persist on DNA, interfering with essential DNA transactions such as replication and transcription; the resulting genomic instability is proposed to accelerate leaf senescence.

Table S1. Primers used in this work.

| **Primer** | **Sequence (5’ to 3’)** | **Purpose** |
| --- | --- | --- |
| ACT2_qRT_F | TCCCTCAGCACATTCCAGCAGAT | RT-qPCR |
| ACT2_qRT_R | AACGATTCCTGGACCTGCCTCAT | RT-qPCR |
| WSS1A_qRT_F | ACGCTCATGGTCCTCATAACGC | RT-qPCR |
| WSS1A_qRT_R | ACATCAACTCCTCGCATTCCTTTC | RT-qPCR |
| SUMO3_qRT_F | TTCTCAAGGTCAAGAGCCAGGATG | RT-qPCR |
| SUMO3_qRT_R | TCGGCGGTCGCAGTAAACATAC | RT-qPCR |
| CAB2_qRT_F | GCCTCAACAATGGCTCTCTC | RT-qPCR |
| CAB2_qRT_R | TGGCTTGGCAACAGTCTTC | RT-qPCR |
| CSAP_qRT_F | ACGACCATTAGCTTCGTGCTTCC | RT-qPCR |
| CSAP_qRT_R | ATCGCATTGGCCATGTTTCTCC | RT-qPCR |
| SAG12_qRT_F | GTTAATGATGAGCAAGCACTG | RT-qPCR |
| SAG12_qRT_R | GGAAATCAAAACCACCTCC | RT-qPCR |
| SAG13_qRT_F | CTCATATCCTCTGCTGCTGGAGTC | RT-qPCR |
| SAG13_qRT_R | CCAGCTGATTCATGGCTCCTTTG | RT-qPCR |
| WRKY6_qRT_F | GCAACAGCAACAACAGAACAA | RT-qPCR |
| WRKY6_qRT_R | TGCCTTGGTACTATCGTCTCC | RT-qPCR |
| SAIL_815_C01_LP | ATATTCGCATTGTCGATGAGC | Genotyping |
| SAIL_815_C01_RP | CAATGGAAACATCATGAACCC | Genotyping |
| SUMO3_SM_F | CAGGCCTTCGTATTTCTCACAG | Genotyping |
| SUMO3_SM_R | GCATATAAGACCACTGACGCTG | Genotyping |
| WSS1A_YC_F | GCAGAGCTCACCATGAGTTCGGCGAATTTGG | Yeast complementation |
| WSS1A_YC_R | TATGCGGCCGCTTCAAGTGCCGACATTAGGAG | Yeast complementation |
| WSS1B_YC_R | GCAGAGCTCACCATGGTCTCTCATCGCATGG | Yeast complementation |
| WSS1B_YC_R | TATGCGGCCGCTCTAGGAACCTGTGGGGTGTG | Yeast complementation |
| WSS1A_C_F | GAGTTCACTAGTATGAGTTCGGCGAATTTGGAAG | Cloning |
| WSS1A_C_R | GTCTCTAGAAGGCCTAGTGCCGACATTAGGAGCACCAG | Cloning |
| WSS1A∆C_C_F | GTCTCTAGAGGATCCATGAGTTCGGCGAATTTGGAAG | Cloning |
| WSS1A∆C_C_R | GTCTCTAGAGCGGCCGCTAGTGCCGACATTAGGAGCACCAG | Cloning |
| SUMO3_C_F | CTGCCCGGGACTAGTATGTCTAACCCTCAAGATGACAAG | Cloning |
| SUMO3_C_R | GTCTCTAGAAGGCCTAAGCCCATTATGATCGAAAAGCA | Cloning |
| WSS1A^mtSIM^_SDM_F | AACGTCTCGTGATGCGACTGAAGAATCTTTTG | Site-directed mutagenesis |
| WSS1A^mtSIM^_SDM_R | AACGTCTCGATCAGCGGCATCTGATCCCTGTTG | Site-directed mutagenesis |
| WSS1A^mtSUMO^_SDM_F | CCACAAGCCTCACATTTCGCTAACCTCACCTCATTTTCAAGCG | Site-directed mutagenesis |
| WSS1A^mtSUMO^_SDM_R | CGCTTGAAAATGAGGTGAGGTTAGCGAAATGTGAGGCTTGTGG | Site-directed mutagenesis |
| SUMO3AA_SDM_F | GATTTGCTCGTAGAGCAGCACTCATCGCCCGG | Site-directed mutagenesis |
| SUMO3AA_SDM_R | CCGGGCGATGAGTGCTGCTCTACGAGCAAATC | Site-directed mutagenesis |
| NTS-IDR_F | TCCACAGATGCTTTAGAAGATGAAGAGAAT | Cloning |
| NTS-IDR _R | ACTAGTCTGCAGAAGGGCGAA | Cloning |
| NTS-IDR_ F | CGCCCTTCTGCAGACTAGTATGAGTTCGGCGAATTTGGAAGATT | Cloning |
| NTS_IDR_ R | CTTCATCTTCTAAAGCATCTGTGGATGGGCAGAATTCAGAGAGA  AGCTTG | Cloning |
| NTS_C_F | ACTAGTATGAGTTCGGCGAATTTGGA | Cloning |
| **Primer** | **Sequence (5’ to 3’)** | **Purpose** |
| NTS_C_R | AGGAGGCCTTGGGCAGAATTCAGAGAGAAGC | Cloning |
| IDR_ATG_C_F | AGAACTAGTATGTCCACAGATGCTTTAGAAGATGAA | Cloning |
| IDR_C_R | AGGAGGCCTTTGGTTTGCATTGTAACTCGTCG | Cloning |
| WSS1A_pMal_F | GAGTTCGCGGCCGCATGAGTTCGGCGAATTTGGAAG | Protein purification |
| WSS1A_pMal_R | GAGTTCGGATCCTCAAGTGCCGACATTAGGAGCACCAG | Protein purification |
| eGFP_pMal_F | GAGTTCGCGGCCGCATGGTGAGCAAGGGCGAG | Protein purification |
| eGFP_pMal_R | GAGTTCGGATCCTTACTTGTACAGCTCGTCCATGC | Protein purification |
